# Daily Rhythmicity of Clock Gene Transcripts in Atlantic Cod Fast Skeletal Muscle

**DOI:** 10.1371/journal.pone.0099172

**Published:** 2014-06-12

**Authors:** Carlo C. Lazado, Hiruni P. S. Kumaratunga, Kazue Nagasawa, Igor Babiak, Alessia Giannetto, Jorge M. O. Fernandes

**Affiliations:** 1 Faculty of Biosciences and Aquaculture, University of Nordland, Bodø, Norway; 2 Department of Biological and Environmental Sciences, University of Messina, Messina, Italy; University of Lübeck, Germany

## Abstract

The classical notion of a centralized clock that governs circadian rhythmicity has been challenged with the discovery of peripheral oscillators that enable organisms to cope with daily changes in their environment. The present study aimed to identify the molecular clock components in Atlantic cod (*Gadus morhua*) and to investigate their daily gene expression in fast skeletal muscle. Atlantic cod clock genes were closely related to their orthologs in teleosts and tetrapods. Synteny was conserved to varying degrees in the majority of the 18 clock genes examined. In particular, *aryl hydrocarbon receptor nuclear translocator-like 2* (*arntl2), RAR-related orphan receptor A* (*rora*) and *timeless* (*tim*) displayed high degrees of conservation. Expression profiling during the early ontogenesis revealed that some transcripts were maternally transferred, namely *arntl2*, *cryptochrome 1b* and *2* (*cry1b* and *cry2*), and *period 2a* and *2b* (*per2a* and *per2b*). Most clock genes were ubiquitously expressed in various tissues, suggesting the possible existence of multiple peripheral clock systems in Atlantic cod. In particular, they were all detected in fast skeletal muscle, with the exception of neuronal PAS (Per-Arnt-Single-minded) domain-containing protein (*npas1*) and *rora*. Rhythmicity analysis revealed 8 clock genes with daily rhythmic expression, namely *arntl2*, circadian locomotor output cycles kaput (*clock*), *npas2*, *cry2*, *cry3 per2a*, nuclear receptor subfamily 1, group D, member 1 (*nr1d1*), and *nr1d2a*. Transcript levels of the myogenic genes *myogenic factor 5* (*myf5*) and *muscleblind-like 1* (*mbnl1*) strongly correlated with clock gene expression. This is the first study to unravel the molecular components of peripheral clocks in Atlantic cod. Taken together, our data suggest that the putative clock system in fast skeletal muscle of Atlantic cod has regulatory implications on muscle physiology, particularly in the expression of genes related to myogenesis.

## Introduction

Circadian clocks are ubiquitous time-keeping systems found in a wide range of organisms, enabling them to adjust to light (L) - dark (D) cycles over a 24 h period [1]. It is not surprising that circadian clocks are evolutionarily conserved [2], [3] since synchronization of behavioral, physiological and metabolic processes to cyclic environmental factors represent a significant advantage [4]. The core machinery of the circadian clock consists of intracellular transcriptional-translational feedback loops regulated by clock genes and corresponding proteins [1]. This regulatory network is composed of two main arms: the transcriptional activation (positive) and the repressor (negative feedback) arms [5]. The positive arm of the core clock is formed by two members: circadian locomotor output cycles kaput (Clock) and Aryl hydrocarbon receptor nuclear translocator-like (Arntl1 or Bmal1). They are basic-helix-loop-helix (bHLH) transcription factors that heterodimerize via a PAS domain in the cytosol [6]. The Clock:Arntl1 heterodimer binds to E box (CACGTG) enhancer elements in the promoters of genes encoding *period* (*per*) and *cryptochrome* (*cry*) in the negative arm of the transcriptional-translational loop. In the negative feedback loop, Per and Cry proteins block the action of Clock:Arntl1 and thereby inhibit their transcription. However, in some organisms either Clock or Npas2 proteins form heterodimers with Arntl proteins to activate transcription (Clock:Arntl1 and Npas2:Arntl1) [7]. In *Drosophila*, TIM and PER form heterodimers that act as negative regulators of the CLOCK-BMAL1 complex [8]. In the stabilizing loop, Clock:Arntl1 activates the orphan nuclear receptors Rev-erb α/β and RAR-related orphan receptor alpha (Rora). These genes are part of the core clock, as they link the feedback loops through their proteins that repress or activate *arntl1* expression. This autoregulatory mechanism results in a cyclic, self-sustained expression of clock genes with an approximately 24-h period [1].

In teleosts, clock genes have been studied mainly in model species such as zebrafish (*Danio rerio*), tiger pufferfish (*Takifugu rubripes*), spotted green pufferfish (*Tetraodon nigrovirides*), Japanese medaka (*Oryzias latipes*) and the three-spined stickleback (*Gasterosteus aculeatus*) [1], [23], [9]. The molecular components of the clock system in a number of aquaculture fish species, such as in European sea bass (*Dicentrarchus labrax*) [10], Senegalese sole (*Solea senegalensis*) [11], rainbow trout (*Oncorhynchus mykiss*) [12] and Atlantic salmon (*Salmo salar*) [13], have also been studied to a limited extent. he Somalian cavefish (*Phreatichthys andruzzii*) provided significant new insights into the evolution and entrainment of circadian clocks. In spite of being blind, this cavefish still retains a clock that responds to food rather than light [14]. A recent study in medaka has demonstrated that the onset of clock rhythmicity can be manipulated by accelerating development through elevated temperature or by artificially removing the choriont [9].

The central clock in teleosts is believed to be located in the pineal gland (or retina) [15]. However, clock genes are not only confined to these central pacemakers but they are also present in various tissues, thus implying the possible existence of peripheral circadian oscillators [16]. Circadian oscillators discovered in peripheral tissues challenge the hypothesis that circadian rhythmicity is controlled exclusively by the central clock machinery. In fact, it has been demonstrated that peripheral clocks have an integral role in a specific tissue or cell as they drive the circadian expression of specific genes involved in various physiological processes [17]. The study of peripheral oscillators has been of particular significance in fish because some peripheral tissues are photosensitive and the circadian clocks present within individual cells can be directly entrained by light [18]. In spite of their importance in driving the circadian expression of specific genes involved in muscle physiology [6], [19], [20], peripheral clocks in skeletal muscle have received scant attention in fish, with the exception of a single study in zebrafish [21]. Atlantic cod (*Gadus morhua*) is a particularly interesting species to study circadian rhythms in skeletal muscle, since their growth is markedly influenced by photoperiod conditions. Continuous illumination significantly promotes body growth [22] and modulates the expression of myogenic regulatory factors in fast skeletal muscle of Atlantic cod [23]. The existence of a peripheral clock in Atlantic cod skeletal muscle is plausible and it could well influence myogenesis. It has been shown in mice that *myoD*, a well-known skeletal muscle-specific transcription factor which regulates the myogenic program, is directly regulated by *Clock* and *Bmal1* [19].

The goal of this study was to characterize the molecular components of the circadian clock system in Atlantic cod fast skeletal muscle. Eighteen putative clock genes have been cloned and characterized in this study. Their expression was determined during a daily cycle (12L:12D) and correlated to the expression of muscle-related genes.

## Materials and Methods

### Animal ethics

All fish handling procedures in this experiment were in accordance to the guidelines set by the National Animal Research Authority (Forsøksdyrutvalget, Norway) and have been approved by the ethics committee of the Faculty of Biosciences and Aquaculture of the University of Nordland, Norway.

### Molecular cloning of Atlantic cod clock genes

Previously characterized clock genes from several species were used to identify the clock genes of Atlantic cod by homology cloning. To amplify the cDNA fragments, gene specific primers were designed either from contigs of the assembled expressed sequence tags obtained from the National Center for Biotechnology Information (NCBI, www.ncbi.nlm-nih.gov) database or the Ensembl Atlantic cod genome assembly (www.ensembl.org; gadMor1).

Partial gene sequences were amplified using pooled cDNA (brain, pituitary and muscle) derived from three adult individuals (sex undetermined) following a PCR amplification protocol involving 40 cycles of denaturation (30 s at 94°C), annealing (30 s at 60°C), and extension (1 min at 72°C). The resulting PCR products were cloned and sequenced as detailed elsewhere [24]. The identity of the amplified cDNA fragment was confirmed by BLAST similarity searches at NCBI.

### Phylogenetic and synteny analyses of Atlantic cod clock genes

Partial sequences of Atlantic cod clock genes were used as probes for *in silico* cloning using the Atlantic cod genome assembly available at Ensembl, in order to obtain longer sequences for phylogenetic analysis. Deduced amino acid sequences were aligned with their corresponding orthologs in various species using MUSCLE Multiple Sequence Alignment (www.ebi.ac.uk/Tools/msa/muscle). The aligned sequences were further fine-tuned by eliminating gaps and highly divergent regions with Gblocks 0.9lb (molevol.cmima.csic.es). The resulting trimmed aligned sequences were used for phylogenetic analysis in PhyML 3.0 (www.atgc-montpellier.fr/phyml). Likelihood analysis was performed using the LG substitution model with four substitution rate categories and an estimated γ shape parameter. Branch support was calculated by aLRT SH-like tests. TreeDyn (TreeDyn.org) was used to view the resulting radiation trees. Synteny analyses were performed in Genomicus v64.01 (www.dyogen.ens.fr/genomicus-64.01). The synteny maps included all teleost species available in the browser. Clock genes not yet annotated in Genomicus were identified in Ensembl and synteny maps were manually constructed.

### Daily rhythm experiment

Atlantic cod juveniles weighing 100.0±6.0 g (mean ± standard deviation [SD]) were stocked in 250 m^3^ tanks at a density of 60 individuals per tank. To ensure minimal disturbance of the fish during sampling, each tank was exclusively dedicated to a single sampling point. Prior to the experiment, the fish were acclimated for at least 3 weeks under a 12L:12D daily photoperiod regime. Water parameters were monitored and maintained as follows: temperature at 7.0±0.6°C (mean ± SD) and dissolved oxygen at 89±2.8%. A commercially formulated diet (Amber Neptun, Skretting AS, Stavanger, Norway) was supplied at a daily ration of 5% (w/w) of the fish body weight. Fluorescent white light tubes (Aura Light International AB, Karlskrona, Sweden) were used to provide illumination and light intensity was measured during a 24-h cycle with a Hanna Hai 97500 Luxmeter (Hanna Instruments, Kungsbacka, Sweden). Tissue sampling was performed at 3-hour intervals (Zeitgeber time: ZT0, 3, 6, 9, 12, 15, 18, 20 and 24) for a period of 24 h. Samples taken at ZT0 were collected immediately after the light reached its maximum intensity (120 lux), while those at ZT24 were collected just before the gradual transition to the light phase, with an intensity increase of 2 lux·min^−1^ over 60 mins. There was an approximate 30 min transition time (ZT12), in which the light intensity decreased at a rate of 4 lux·min^−1^ to total darkness between the light (ZT0-9) and dark (ZT15-24) phases. ZT12 samples were collected during this transition period. Ten fish were taken at each sampling point and killed by immersion in seawater containing 0.5 g·L^−1^ tricaine methanesulfonate (Sigma, Oslo, Norway). Collection of samples during the dark phase was conducted in a room with white light intensity not greater than 1 lux and it was ensured that exposure of an anesthetized fish to these conditions did not last longer than 5 min. Fast skeletal muscle was taken from the area below the second dorsal fin, skin was removed and the tissue was washed with cold, sterile 1× PBS to remove contaminating blood before immersion in liquid nitrogen. Samples were stored at −80°C until RNA extraction. Blood was also collected from the caudal vein for melatonin quantification. Heparinized blood samples were centrifuged at 1,200×g for 15 min at 4°C. Thereafter, the plasma was collected and kept at −80°C until further analysis.

### Plasma melatonin assay

Plasma melatonin was quantified by competitive ELISA with a commercially available Melatonin ELISA kit (IBL, Hamburg, Germany). This kit has been previously validated with fish plasma melatonin and had a method specificity of nearly 100% [25].

### Tissue and early ontogeny sample collection

Tissue samples from adult Atlantic cod previously collected in a sister study [23] were used to determine the expression of clock genes in various tissues and organs, namely blood, liver, spleen, stomach, mid-intestine, kidney, brain, pituitary, heart, gills, eye, dorsal skin, ventral skin, testis, ovary, and muscle. Fish were reared under natural photoperiod conditions (NL) for Bodø, Norway (67°N, 14°E) and sacrificed as above on week 45 (sunrise at approximately 8:08 AM and sunset: 15:23 PM). All tissue samples were collected between 9 am and 12 noon.

Samples used for early ontogenetic expression of clock genes were previously collected in a separate study [26]. The fertilized egg defined the onset of ontogenesis and was denoted as t = 0. The various developmental stages were confirmed by visualization under an optical microscope and the following samples were collected: unfertilized egg, 1-cell (5.2 hour post-fertilization at 7°C, hpf), 2-cell (6.8 hpf), 16-cell (13.5 hpf), oblong (31 hpf), germ ring (58 hpf), 50% epiboly (98 hpf), 10 somite (119 hpf), golden eye (225 hpf), hind gut (304 hpf), 1st feeding (346 hpf), and 20 days post-first feeding (828 hpf). The photoperiod regime was constant darkness (DD, 0L:24D) from unfertilized egg to hind gut stage and thereafter shifted to constant illumination (LL, 24L:0D, white light with an intensity of approximately 80 lux).

### Gene expression analyses

Total RNA was extracted from the tissue samples using the *mir*Vana miRNA Isolation Kit (Ambion, Oslo, Norway). The RNA was then quantified with a Nanodrop ND-1000 Spectrophotometer (Thermoscientific, CO, USA) and its quality assessed by denaturing electrophoresis on a 1.2% (w/v) agarose gel. cDNA was synthesized from 1 µg/mL total RNA by QuantiTect Reverse Transcription kit (Qiagen, Nydalen, Sweden).

All primers used in the expression studies were designed with PerlPrimer (www.perlprimer.sourceforge.net) across intron/exon borders to avoid amplification of contaminating genomic DNA, as reported [27]. Expression of the clock genes in different tissues and during early ontogeny was determined by semi-quantitative PCR (RT-PCR) using the primers given in Table S1. Thermocycling parameters were 94°C for 3 min, followed by 30 cycles of 30 s at 94°C, 30 s at identified annealing temperature for each primer set (Table S1), and 30 s at 72°C, with a final elongation step of 72°C for 3 min. PCR products were visualized by electrophoresis on a 1.0% (w/v) agarose gel and photographed with a Kodak gel documentation system v.1.0.5 (Oslo, Norway). *Elongation factor 1-alpha 1 (eef1a)* was used as an internal reference in tissue distribution analysis. On the other hand, luciferase (*luc*) was used as internal control in the expression of clock genes during early stages of ontogenesis because it was previously shown in zebrafish that most housekeeping genes are not stable during this period, particularly during maternal-zygotic transition [27], [28].

Quantification of clock gene transcripts in the fast muscle during a daily cycle was performed by real-time PCR (qPCR) using the primers indicated in Table S1 and the LightCycler 489 SYBR Green I master chemistry (Roche, Basel, Switzerland) on a LightCycler 480 (Roche) thermocycler. Diluted cDNA was run in duplicate, including minus reverse transcriptase and no template controls. The qPCR reaction was performed according to this thermocycling protocol: initial denaturation at 95°C for 15 min, followed by 45 cycles of 15 s at 94°C, 20 s at defined annealing temperature (Table S1) for each primer set and 20 s at 72°C. In order to calculate amplification efficiencies, a five-point standard curve of 2-fold dilution series were prepared from pooled cDNA. Cycle threshold (*C_T_*) values were calculated with the LightCycler 480 software using a fluorescence arbitrary value of 1. To validate that the primers were amplifying the gene of interest, PCR products were verified by Sanger sequencing. In order to determine if there was an expressional correlation between clock and muscle-related genes (Table S2), transcript levels of selected muscle-related genes were also quantified in fast muscle during a daily cycle with the same PCR protocol.

Three reference genes (Table S2), namely *acidic ribosomal protein* (*arp*), *ubiquitin* (*ubi*) and *eef1a* were tested for their suitability for normalization of the experimental data using the geNorm algorithm, as reported [27]. Expression of target genes was normalized using the geometric average of *arp* and *ubi*, for they were the most stable on this given experimental setup.

### Statistical analyses

Differences in clock gene expression between time points were analyzed with the SigmaStat statistical package (Systat software, London, UK). A one-way ANOVA was performed on data sets that passed the tests of normality and equal variance, and Tukey's multiple comparison test followed to delineate differences between time points. For data sets that did not follow a Gaussian distribution or did not meet the equal variance requirements, Kruskal-Wallis one-way ANOVA on ranks followed by Dunn's multiple comparison test were used instead. The level of significance was set at *P*<0.05.

To evaluate daily rhythmicity in the expression of the clock genes and muscle-related genes, COSINOR analysis was performed by fitting a periodic sinusoidal function to the expression values of genes across the nine time points, according to the formula *ƒ (t)* = M+ Acos(*t*/*pi*/12 – φ), where *ƒ (t)* is the gene expression level at a given time, mesor (M) is the mean value, A is the sinusoidal amplitude of oscillation, *t* is time in hours and φ is the acrophase (peak time of the approximating sinusoidal function). The statistical significance *P* of the approximated 24 h waveform was defined by the noise/signal of the amplitude (SE(A)/A) and expression was considered to display daily rhythmicity if *P*<0.3 [29]. Correlation analysis between clock and muscle-related gene expression was done using the Pearson's correlation test (*r*).

## Results

### Atlantic cod clock genes and their evolutionary characteristics

Partial sequences of 18 clock genes from both loops of the clock machinery were cloned in Atlantic cod: *arntl1* (*bmal1*, Genbank accession JN643704), *arntl2* (*bmal2*, JX035864), *clock* (JN643707), *cry1a* (JN643708), *cry1b* (KC204818), *cry-dash* (KC204819), *cry2* (JN643709), *cry3* (JX035865), *npas1* (KC204820), *npas2* (JX035866), *nr1d1* (*rev-erbα*, KC204822), *nr1d2a* (*rev-erbβa*, JN643712), *nr1d2b* (*rev-erbβb*, KC204823), *per1* (JN643710), *per2a* (JN643711), *per2b* (JX035863), *timeless* (*tim*) (JN634817) and *rora* (KC204821).

**Figure 1:**
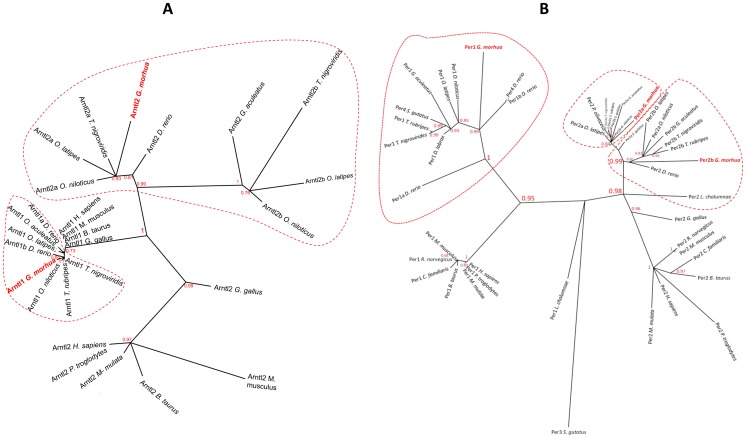
Radiation trees of representative clock genes from the transcriptional activation (A: *arntl*) and repression (B: *per*) arms of the Atlantic cod molecular clock system. The unrooted trees were constructed by maximum likelihood analysis using an LG substitution model with four substitution rate categories. Branch support was calculated by aLRT SH-like tests. Orthologous genes from teleosts and tetrapods were used in the construction of the phylogenetic trees. Atlantic cod clock genes cloned in the present study are highlighted in red bold font. Teleostean clades are circled by a dotted red line. Phylogenetic trees of other gene families are provided in Figure S1A-I.

Teleost *arntl*s formed two distinct clades. The fish *arntl1* group was closely related to their tetrapod orthologs with short branch lengths (Fig. 1A). Three clusters of *arntl2* were observed: piscine *arntl2a* including *G. morhua arntl2*, piscine *arntl2b* and tetrapod *arntl2*. The fish *arntl2a* clade containing *G. morhua arntl2* also included orthologs from *Oreochromis niloticus*, *O. latipes* and *T. nigroviridis*. The three paralogs of Atlantic cod *per* genes were found in three clusters (Fig. 1B). *G. morhua per1* was closely related with its orthologs in *D. rerio*, *O. latipes* and *O. niloticus*. The two Atlantic cod *per2* genes clustered in two separate groups, along with other teleost orthologs, such *G. aculeatus, O. latipes, O. niloticus, T. nigroviridis, T. rubripes* and *Paralicthys olivaceus*. The other clock genes found in Atlantic cod were also closely related to their teleost and other vertebrate orthologs (Fig. S1A-I).

**Figure 2:**
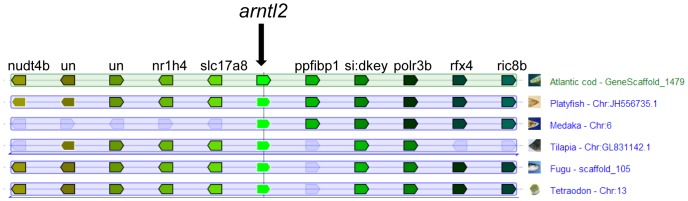
Partial synteny map of *arntl2* of Atlantic cod showing high syntenic conservation. Orthologous genes in *G. morhua*, *O. latipes*, *T. rubripes*, *T. nigroviridis*, *O. niloticus* and *X. maculatus*, are indicated by block arrows showing their position and orientation. Additional synteny results can be found in Figure S2A-O. (un: unidentified gene).

Comparative analysis of the genes surrounding each clock paralog revealed the conservation of synteny amongst teleosts, namely in *D. rerio, G. aculeatus, O. latipes, O. niloticus*, *T. nigroviridis, T. rubripes* and *Xiphophorus maculatus* (Fig. S2A-I). Synteny was not conserved in the genomic regions containing *cry1b* and *nr1d2b* but could be clearly observed for *arntl2*, *rora* and *tim*. The representative partial synteny map for *arntl2* shown in Fig. 2 revealed high conservation, with the presence of several genes (*nudt4b, nr1h4, scl17a8, pfibp1, si:dkey, polr3b, rfx* and *ric8b*) in the same genomic location and orientation as in other teleost species. Nevertheless, this synteny profile was less conserved in medaka.

### Early ontogenetic expression of the clock genes

**Figure 3:**
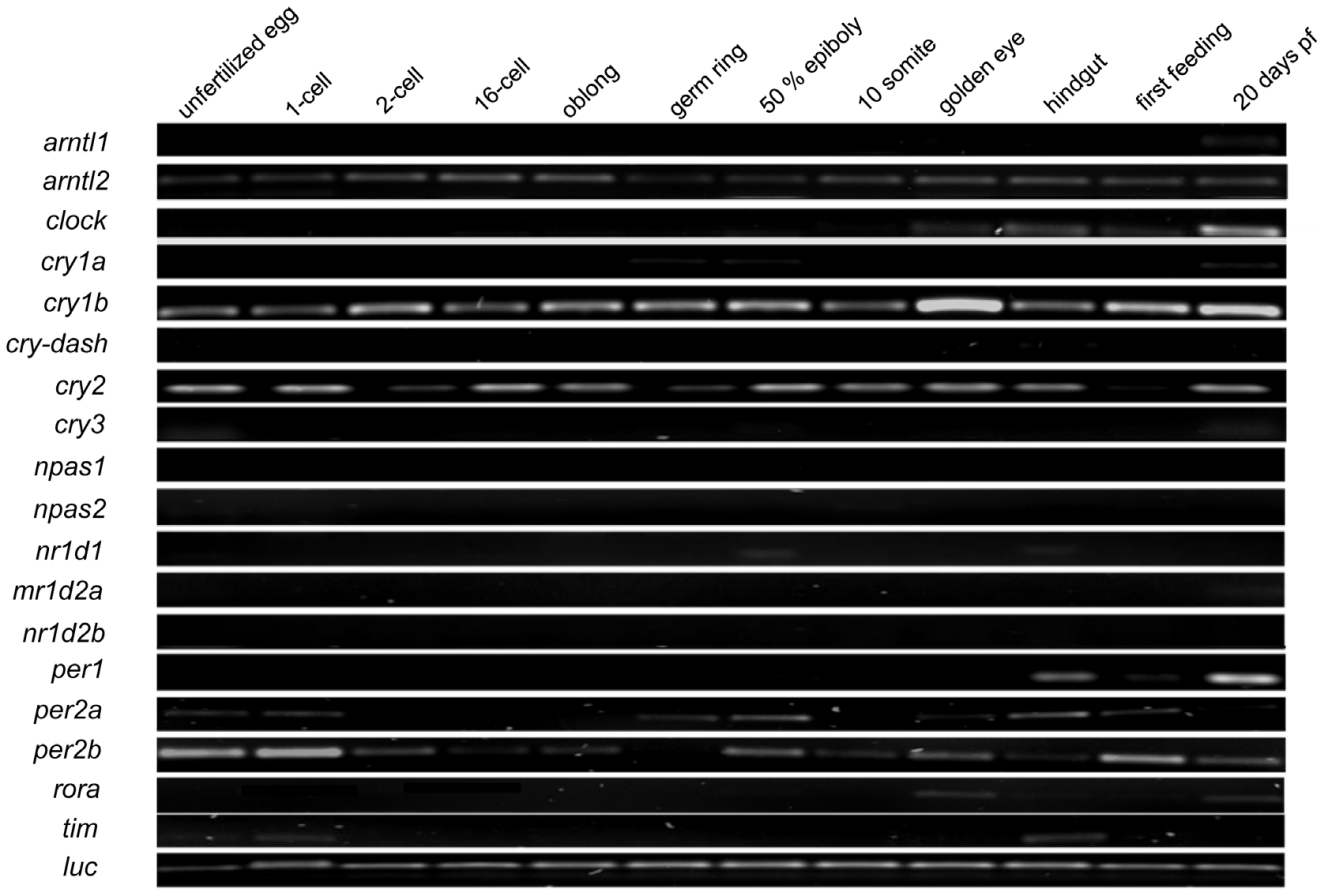
Early ontogenetic expression of clock genes in Atlantic cod. The cDNAs used in semi-quantitative RT-PCR were from the pooled RNA of approximately 50 specimens from each stage [26]. *Luciferase* (*luc*) was used as an external reference.

The various molecular components of the circadian clock system in Atlantic cod were first detected at different ontogeny stages (Fig. 3). *arntl2*, *cry1b* and *cry2* transcripts were maternally transferred and detected in all stages of early development examined, albeit at different levels. *Per2a* and *per2b* were also maternal transcripts but their levels gradually decreased as maternal-zygotic transition progressed; these genes were detected again during the latter part of the development in a stage-dependent manner. Some clock gene transcripts were only detected during the latter part of the development. For instance, *clock* and *rora* started to be transcribed only at the golden eye stage, whereas *arntl1* and *cry1a* mRNAs were only found at 20 days post-first feeding. The two *npas* paralogs as well as *cry3*, *nr1d2a* and *nr1d2b* could not be detected during early ontogeny.

### Presence of clock gene transcripts in different peripheral tissues

**Figure 4:**
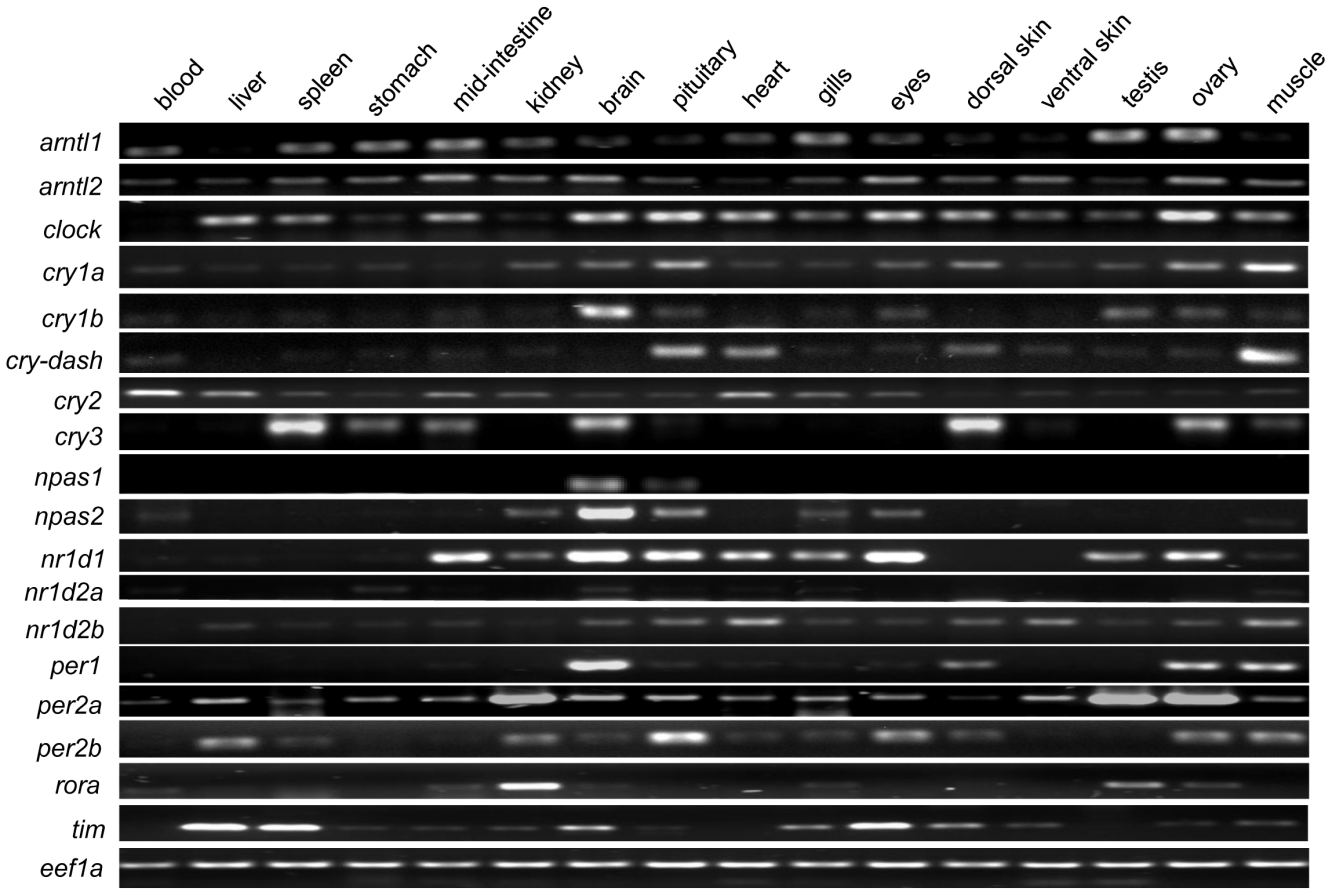
Presence of clock gene transcripts in different tissues of Atlantic cod. Expression of clock genes in various tissues (blood, liver, spleen, mid-intestine, kidney, brain, pituitary, heart, gills, eyes, dorsal skin, ventral skin, testis, ovary, and fast muscle) was analyzed by semi-quantitative RT-PCR. *Eef1a* gene was used as an endogenous reference.

Clock gene transcripts were found in multiple tissues of adult Atlantic cod and showed some tissue specificity in their expression, except *arntl2, cry2* and *per2a*, where ubiquitous expression was observed in all tissues studied (Fig. 4). With the exception of *npas1* and *rora*, all clock genes were detected at varying levels in fast skeletal muscle. The majority of clock genes were highly expressed in brain, with the exception of *cry-dash*. The clock genes *arntl2*, *clock*, *per2a, per2b* and *nr1d1* were prominent in organs related to the endocrine system (e.g. pituitary) and light perception (e.g. eyes). Apparent differences were observed in expression patterns between paralogs. For example, *cry1a* was found in 13 out of the 18 tissues while *cry1b* was only present in 11 tissues. Similarly, *per2a* was detected in all tissues while *per2b* was only found in 11 tissues. Interestingly, the majority of clock genes was present at a higher level in dorsal rather than in ventral skin. Regardless of sex, *arntl1, clock, per2a* and *nr1d1* were detected abundantly in the gonads. On the other hand, *cry3, per1, per2b* and *tim* transcripts were only found in ovary but not in testis.

### Rhythmicity of clock gene transcription during a daily cycle

**Figure 5:**
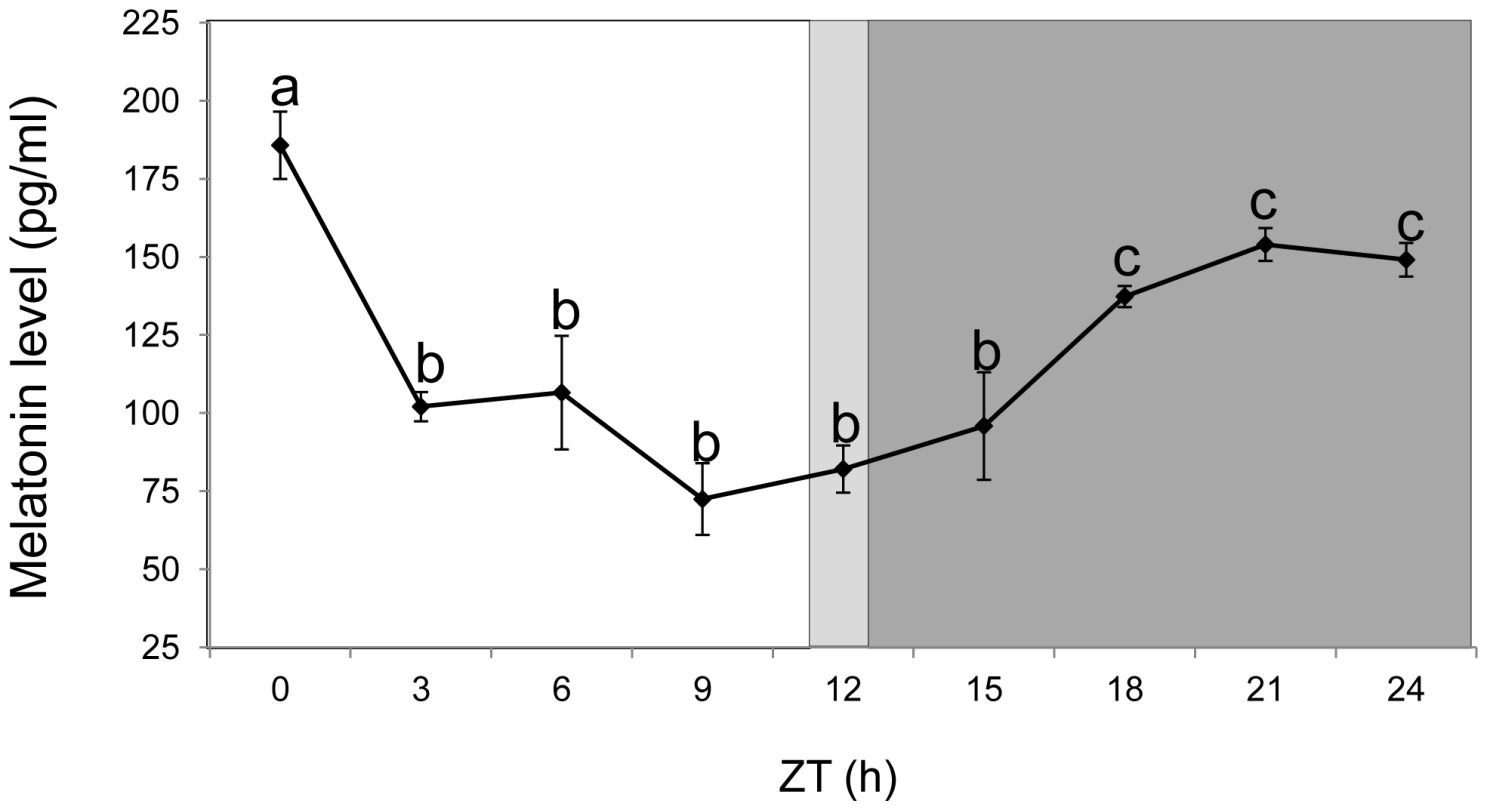
Changes in the plasma melatonin level during a daily cycle. Plasma melatonin levels were quantified by competitive ELISA. Values shown are mean+SEM of plasma melatonin (n = 6). The background represents the photoperiod regime: the light background is the light phase, the dark background is the dark phase and light gray indicates the light-dark transition phase.

Plasma melatonin levels showed a biphasic diurnal pattern, with higher concentrations during the dark phase than the light phase (Fig. 5). Its highest level was observed just after the light intensity reached its maximum (ZT0), while the lowest level was noted 3 hours prior to light-dark transition (ZT9). As darkness progressed, the level of melatonin increased throughout the dark phase, reaching a significantly constant level at later hours (ZT18 – ZT24).

**Figure 6:**
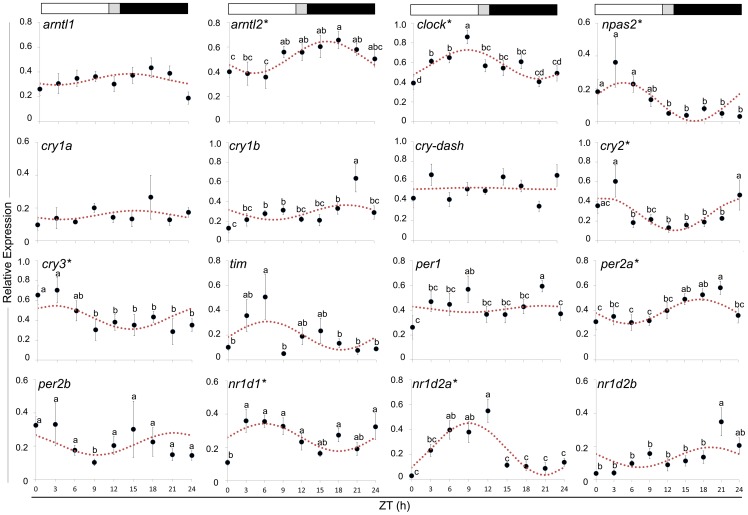
Expression of clock genes in fast skeletal muscle during a daily cycle. The values are mean+SEM (n = 6) of the normalized transcript levels of each clock gene. Statistical difference between time points is indicated by different letter notations. The broken line is the periodic sinusoidal function of gene expression in a circadian cycle constructed from the periodicity parameters calculated by COSINOR. An asterisk (*) besides the gene name indicates significance of daily rhythmicity. The photoperiod regime is represented by the composite block above the graph. White, black and gray represent the light phase, the dark phase and the light-dark transition phase, respectively.

There was no significant difference on the temporal expression of *arntl1* but the *arntl2* paralog displayed daily rhythmic expression (*P* = 0.01) and it was preferentially expressed during the dark phase having an acrophase at ZT 16.1 h. mRNA levels of *clock*, a known co-dimer of *arntl*, displayed daily rhythmicity (*P* = 0.06) although with an acrophase during the light phase (ZT 9.04 h). Expression of *npas2*, an alternative component of the positive arm of the transcriptional feedback loop, showed daily rhythmic expression (*P* = 0.08) with an acrophase at ZT 4.36 h. The rhythmicity parameters of these genes are provided in Table 1. Transcript levels of 3 out of the 9 transcriptional repressors studied (*cry2* (*P* = 0.05), *cry3* (*P* = 0.27) and *per2a* (*P* = 0.07)) displayed daily rhythmic expression (Fig. 6, Table 1). *Cry2* had an acrophase at ZT 1.04 h, which occurred in an inverse phase to *arntl2*. Significant temporal differences and daily rhythmicity were observed in *cry3* transcript levels. *Per2a* was preferentially expressed during the dark phase with the acrophase noted at ZT 17.5 h. Significant temporal differences were observed in *cry1b, tim*, *per1* and *per2b* mRNA levels but these changes were not rhythmic. Two of the three orphan nuclear receptor genes exhibited daily rhythmic expression: *nr1d1* (*P* = 0.21) and *nr1d2a* (*P* = 0.01) (Fig. 6). Both genes had a light phase responsive profile with acrophases at ZT 6.01 h and ZT 9.01 h for *nr1d1* and *nr1d2a*, respectively. Expression of *nr1d2b* was arrhythmic (*P* = 0.51), even if some significant temporal differences were observed.

**Table 1:** Rhythmicity parameters of clock gene transcription in Atlantic cod fast skeletal muscle.

Gene^1^	Peak of expression/Acrophase (h)	*P*
*arntl1*	14.6	0.41
***arntl2***	16.1	0.01
***clock***	9.04	0.06
***npas2***	4.36	0.08
*cry1a*	15.6	0.62
*cry1b*	19.3	0.63
*cry-dash*	10.3	0.99
***cry2***	1.04	0.05
***cry3***	2.53	0.27
*per1*	21.2	0.88
***per2a***	17.5	0.07
*per2b*	21.4	0.83
*tim*	6.33	0.37
***nr1d1***	6.01	0.21
***nr1d2a***	9.01	0.01
*nr1d2b*	19.3	0.51

1 Clock genes whose transcript levels displayed daily rhythmicity are highlighted in bold. The P value is defined as the noise/signal ratio of the oscillation amplitude. P<0.3 and a significant difference with ANOVA (p<0.05) indicate daily rhythmicity.

### Daily expression of muscle-related genes and its correlation with expression of clock components

**Figure 7:**
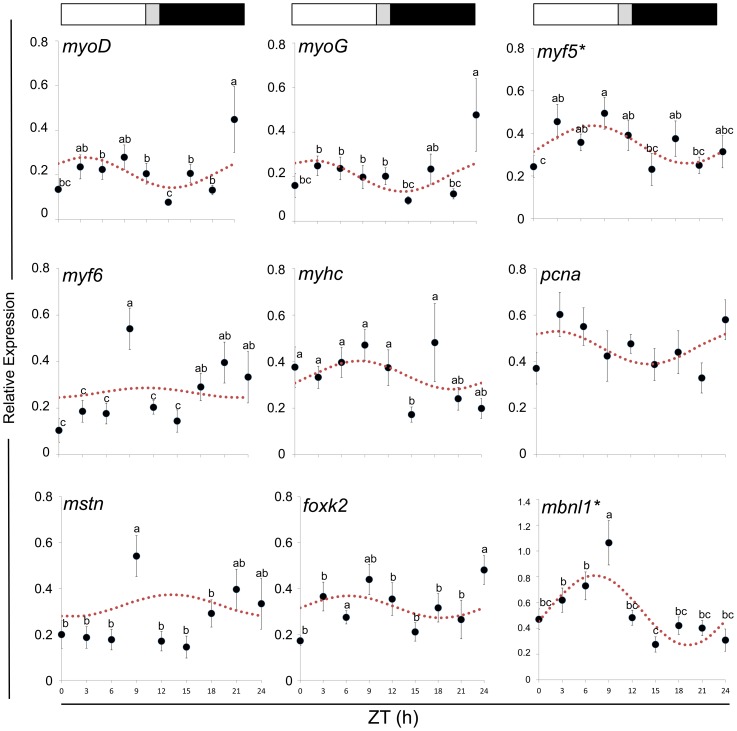
Daily expression of muscle-related genes. The values presented here are mean+SEM (n = 6) of normalized expression. Statistical differences between time points are indicated by different letter notations. The broken line is the periodic sinusoidal function of the gene expression in a daily cycle constructed from the rhythmicity parameters provided in Table S3. An asterisk (*) beside the gene name indicates that the expression is daily rhythmic. The photoperiod regime is represented by the composite block above the graph. White, black and gray represent the light phase, the dark phase and the light-dark transition phase, respectively.

*myf5* (*P* = 0.15) and *mbnl1* (*P* = 0.05) displayed daily rhythmic expression (Fig. 7) with acrophases at ZT 7.5 h and ZT 7.2 h, respectively. Even if other muscle-related genes did not display daily rhythmicity, their expression profiles had expression peaks during the light phase, with the exception of *mstn* (Table S3).

Expression of the clock components showed intra-group and inter-group positive/negative correlations (Table 2). Particularly, there was a strong positive correlation within components of the transcriptional repressor arm specifically in the following gene pairs: *cry3*:*per2b* and *per2a*:*cry1b*. The components of the transcriptional activator arm *arntl1*, *arntl2* and *npas2* showed moderate to strong negative correlation to several members (i.e. *cry2*, *cry3*, *tim* and *per2a*) of the transcriptional repressor arm.

**Table 2:** List of clock gene pairs showing either positive or negative expression correlation in fast muscle during a daily cycle.

Positive correlation			Negative correlation		
Gene	Gene	*r*	Gene	Gene	*r*
***arntl1***	***arntl2***	0.51	***arntl1***	*cry2*	−0.60
***arntl2***	*per2a*	0.64	***arntl2***	***npas2***	−0.78
***clock***	*nr1d1*	0.66	***arntl2***	*cry2*	−0.54
***clock***	*nr1d2a*	0.60	***arntl2***	*cry3*	−0.73
***npas2***	*cry2*	0.58	***arntl2***	*tim*	−0.60
***npas2***	*cry3*	0.83	***npas2***	*per2a*	−0.54
***npas2***	*tim*	0.61	***npas2***	*nr1d2b*	−0.57
*cry2*	*cry3*	0.61	*cry1b*	*cry3*	−0.58
*cry3*	*per2b*	0.76	*per2a*	*cry3*	−0.52
*per2a*	*cry1b*	0.74	*cry3*	*nr1d2b*	−0.74
*per2a*	*nr1d2b*	0.73			

NOTE: Only correlations with *r*>+0.5 or *r*<−0.5 and including at least one gene with significant daily rhythmicity are shown in this table. Underlined genes names indicate that they are part of the stability loop of the transcriptional-translational feedback loop and bold face that they are part of the positive arm. Names of genes involved in the negative arm are neither in bold nor underlined. The following values were set to define the degree of correlation: data are moderately correlated if 0.5<r<0.69 and there is a strong correlation when r ≥0.70.

The patterns of mRNA levels for most muscle-related genes correlated with the daily expression of clock genes (Table 3; Table S4). Transcript levels of *myf5*, *myhc*, *mbnl* and *foxk2* positively correlated with the expression of *clock* with *myf5* and *mbnl1*, showing a strong correlation index (*r*>0.80; Table 3). In addition, both daily rhythmic nuclear receptors positively correlated with *myf5* and *mbnl1* expression. Members of the transcriptional repressor arm (i.e. *per2a*, *per2b*, *cry1b, cry3* and *tim*) showed moderate to strong negative correlation with *pcna, myf6, mstn, myoG, mbnl1* and *foxk2*.

**Table 3:** Correlation of clock and muscle-related transcript levels.

Muscle-related gene	Clock gene							
	*arntl2*	*clock*	*npas2*	*cry2*	*cry3*	*per2a*	*nr1d1a*	*nr1d2a*
*myf5*	−0.10	0.84	0.45	0.17	0.11	−0.47	0.77	0.64
*mbnl1*	−0.28	0.82	0.48	−0.05	0.05	−0.54	0.52	0.55

NOTE: Only genes displaying significant daily rhythmicity are given in this table. The overall correlation profile is given in Table S4. The arbitrary categories that define the degree of correlation are as follows: data are moderately correlated if 0.5<r<0.69 and the correlation is considered strong when r >0.70.

## Discussion

**Figure 8:**
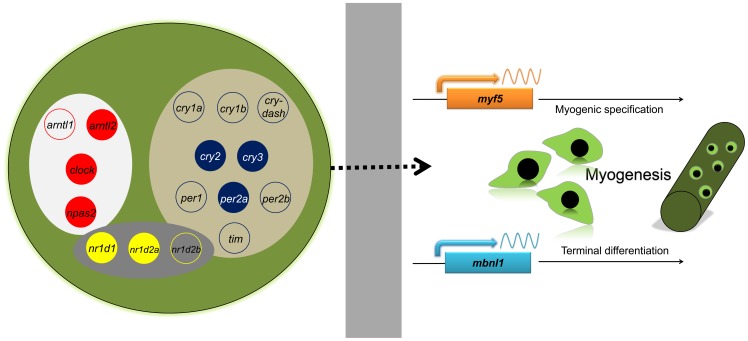
Molecular components of the clock system identified in fast skeletal muscle of Atlantic cod and myogenic genes with daily rhythmic expression. The peripheral clock components in Atlantic cod fast skeletal muscle that have been identified in this study are shown inside the green circle, which represents a muscle fiber. They comprise members of the transcriptional activator arm (in red: *arntl1, arntl2, clock* and *npas2*), transcriptional repressor arm (in blue: c*ry1a, cry1b, cry-dash, cry2, cry3, per1, per2a, per2b* and *tim*) and the stabilizing loop (in yellow: *nr1d1, nr1d2a* and *nr1d2b*). Clock genes with a colored background displayed a daily rhythmic expression in fast skeletal muscle. The daily rhythmicity of *myf5*, a gene for myogenic lineage specification, and *mbnl1*, a gene for terminal muscle differentiation, suggests a possible circadian clock control of myogenesis in Atlantic cod. The grey box indicates that the mechanism underlying this regulatory process remains to be identified.

This is the first report of the molecular components of the circadian clock in Atlantic cod. We have identified 18 clock genes, namely 5 members of the transcriptional activation arm (*arntl1, arntl2, clock, npas1* and *npas2*), 9 genes of the transcriptional repression arm (*cry1a, cry1b, cry-dash, cry2, cry3, per1, per2a*, *per2b* and *tim*) and 4 orphan nuclear receptors of the stabilizing loop (*nr1d1, nr1d2a, nr1d2b* and *rora*). These genes were transcribed in fast skeletal muscle, with the exception of *npas1* and *rora*, suggesting the existence of a possible peripheral clock in this tissue (Fig. 8).

The circadian clock system in fish is more complex than its mammalian counterpart because of the existence of multiple copies of clock genes [1], consistent with our findings in Atlantic cod. The existence of 2 paralogs (i.e. *cry* and *per* genes) per mammalian ortholog could be explained by the teleost-specific third round of the whole genome duplication [30]. In contrast to mammals, which have only 2 copies of *cry* gene [31], teleostean *cry* paralogs constitute a highly divergent family. Gene duplication is a major adaptive genomic response [32] and this phenomenon may lead to the establishment of lineage-specific traits and to the development of novel biological functions [33], [34]. We can speculate that clock genes in Atlantic cod may be sharing similar functions as shown by the strong evolutionary relationship of these clock genes with other teleost species and high syntenic conservation. On the other hand, differences in daily expression of several molecular clock components (discussed below) indicate some degree of divergence and functional diversification. Also, some paralogs (e.g. *cry3, cry1a, nr1d2a*) seem to be evolving much faster than their mammalian orthologs, based on our phylogenetic analysis results. Whether there is a redundancy or uniqueness in the function of each copy of the clock genes in Atlantic cod is yet to be ascertained in further studies. Within teleosts, the circadian clock system has been functionally characterized mostly in zebrafish [1]. The possibility that functional clocks differ across fish species is very likely, since the environment plays a crucial role in circadian regulation.

The detection of clock gene transcripts at stages prior to activation of zygotic transcription indicated that some components of the circadian system of Atlantic cod, specifically *arntl2, cry1b, cry2, per2a*, and *per2b* are of maternal origin. In addition to maternal contribution, environmental stimuli such as light could influence the development of the clock system. The establishment of circadian rhythms clearly requires exposure to environmental zeitgebers such as light and temperature changes [1] and also depends on the developmental stage of the fish [9], [35], [36]. The present study determined the presence of clock components at a specific ontogeny stage but not the onset of rhythmicity of these genes in Atlantic cod. It is noteworthy that *cry1b* was ubiquitously expressed during early Atlantic cod ontogenesis. Cryptochromes are pterine/flavine-containing proteins and play an important role in phototransduction and circadian photosensitivity in *Drosophila* [37]. *cry1b* was prominently expressed at the golden eye stage and the development of this photosensitive organ may have a significant influence in the expression of this gene. In addition to the notable expression of *cry1b*, *clock* and *rora* started to be expressed from the golden eye stage while *per1*, *per2a* and *tim* started to be detected after this stage only. The development of this photoreceptive organ could have a direct influence on the development of several molecular components of the circadian system. Other than light, food could provide a zeitgeber in the entrainment of circadian rhythm and this has already been documented in zebrafish and cavefish [14], [38]. The possible presence of multiple peripheral clocks in Atlantic cod was supported by the ubiquitous expression of clock genes in different tissues and organs. The majority of these genes were expressed distinctively in the eyes and to some extent in the pituitary gland, which was expected since these organs are related to the photo-endocrine axis of the circadian system. The circadian clock system has been regarded as a fundamental mechanism in the photoneuroendocrine regulation of reproduction in fish [39]. The high mRNA levels of clock genes such as *arntl1*, *arntl2*, *clock*, *nr1d1* and *per2a* observed in testes and ovaries imply that these molecular clock genes may influence gonadal physiology in Atlantic cod. Some of the clock genes identified in this study were also differentially regulated in gonads during a maturation cycle in Atlantic cod, thus supporting their possible involvement in sexual maturation (Lazado et al, unpublished data). The several clock genes identified may have immunological implications inAtlantic cod because they were highly expressed in immune-related organs and tissues, such as blood, liver, mid-intestine, gills, kidney, spleen and skin. In fact, it has been shown that the immune response in fish is regulated in a daily or seasonal pattern, and is affected by photoperiod conditions [40], [41], [42].

Recent studies support the notion of a decentralized clock in the understanding of biological rhythms. In particular, the peripheral clocks present in various tissues have been of great interest, as these oscillators are regarded as direct circadian regulators of genes involved in the physiology of specific tissues [17]. Circadian rhythms in tissues and organs are essential to ensure that physiological processes, such as metabolism and cell cycle, undergo at the optimal time as required by the organism. Clock genes are expressed in fast skeletal muscle in several species and the daily rhythmicity observed in a number of these genes provides a possible implication on their essential regulatory functions [6], [19], [21]. Nevertheless, the current knowledge on the relevance of circadian rhythm in fish skeletal muscle physiology is very limited. The two Atlantic cod *arntl* genes had a night-biased expression, since their peaks of expression were observed during the dark phase. This photosensitivity profile is corroborated by the expression of their orthologs in zebrafish skeletal muscle [21] but not in either central or peripheral clocks in rainbow trout [12] and mouse [43]. In the present study, there was no clear relationship between *arntl* and *clock/npas2*, as shown by the weak correlation between these transcripts and the apparent differences in their acrophases. A comparable expression profile was also observed in several peripheral tissues of zebrafish [44]. This antiphase profile revealed that the transcriptional activation arm of the possible clock system in the fast muscle is probably not similar to the established central pacemakers known in other fish species and Arntl may have another co-dimer in the activation arm of the clock system in fast skeletal muscle. In mammals, Bmal1 (Arntl1) has the ability to interact with other bHLH-PAS factors besides Clock [45], [46]. A similar mechanism may be present in Atlantic cod fast skeletal muscle but our study was limited to the transcriptional level.

Among the 5 *cry* genes identified in Atlantic cod, their expression peaks indicated that *cry2* and *cry3* are light-biased whereas *cry1b* is dark-biased. The light-biased expression of *cry2* was similar in peripheral clocks of other teleosts, such as European seabass (*Dicentrarchus labrax*) [47]. Only *cry2* and *cry3* had daily rhythmic expression in Atlantic cod fast skeletal muscle. Both genes displayed moderate to strong negative correlation with *arntl* genes and their expression was in inverse phase with *arntl* as well. These patterns suggest that *cry2* and *cry3* may have a significant function in this arm of the transcriptional feedback loop in skeletal muscle. There was also a moderate positive correlation between *cry2* and *cry3*, suggesting that there may be a tight co-regulatory control between these 2 paralogs in the transcriptional repression arm.

*Cry-dash* and *tim* are two newly-characterized clock-gene homologs and their involvement in the piscine clock system is yet to be identified. Nevertheless, *cry-dash* has been shown to be light inducible in zebrafish [48]. The dark-biased expression of the 3 *period* genes was in agreement with the expression of other teleost orthologs in central and peripheral tissues such as in goldfish (*Carassius auratus*) [29], European sea bass [10] and zebrafish [19], [38]. On the other hand, this circadian preference is not similar to what has been observed in central and peripheral clocks of Senegalese sole [11]. In the established circadian feedback loop model, the proteins translated by *per* and *cry* genes interact to inhibit the transcriptional activation arm [1]. *Per2a*, the only *period* gene with daily rhythmic expression in the present study, did not show a positive correlation with any of the *cry* genes with daily rhythmic expression. However, the transcript levels of Atlantic cod *per2a* showed a strong positive correlation with the arrhythmically expressed *cry1b*. Also, there was an inverse correlation between transcript levels of Atlantic cod *per2b* and *clock*/*npas2*, two genes with circadian rhythmicity in the activation arm. In mammals, Clock has a constitutive expression while Bmal displays daily rhythmic variations in the suprachiasmatic nucleus [49]. Such a mechanism suggests that some genes with arrhythmic expression could still be important components of the peripheral clock in Atlantic cod muscle.

Nuclear receptors such as *nr1d1*, *nr1d2* and *rora* are modulators in the stabilizing loop of the circadian mechanism [1]. Unlike a previous report in zebrafish [21], *rora* was not detected in Atlantic cod fast skeletal muscle but two orphan nuclear receptor genes, *nr1d1* and *nr1d2a*, had daily rhythmic expression with preference towards the light phase. This expression profile was inversely related to those of *arntl* paralogs, in agreement to the known inverse relationship between these genes [43]. *clock* and *npas2* were also suggested in mammals to be regulated by these nuclear receptors in a repressive manner [50], [51]. In the present study, the expression profile of *clock* and *npas2* had a similar trend with the nuclear receptors, and *clock* even showed a positive correlation with *nr1d1* and *nr1d2a*. We did not explore to what extent these nuclear receptors render feedback stability on the circadian clock transcriptional-translational network. Nevertheless, the rhythmicity observed in their expression is a possible indication of their essential role in the circadian rhythm of fast skeletal muscle in Atlantic cod.

Most of the muscle-related genes studied displayed temporal changes in transcript levels during the daily cycle, but only *myf5* and *mbnl1* demonstrated daily rhythmic expression. Such changes imply that muscle physiology is influenced by the light-dark cycle and could be under the regulation of clock genes. It has been demonstrated that the Clock/Bmal1 circadian regulation of MyoD is critical in maintaining skeletal muscle phenotype and function in mice [19]. However, *myoD* in Atlantic cod skeletal muscle did not show daily rhythmicity, as observed in zebrafish [21]. It is plausible that the circadian regulation of piscine *myoD* is not under the same mechanism reported in mouse. Other myogenic regulatory factors might be playing the circadian clock-related transcriptional control of myogenesis in fish, such as the daily rhythmicity of *myf6* transcript levels in zebrafish [21] and *myf5* in Atlantic cod. Higher transcript levels of most myogenic genes analyzed in this study were observed during the light phase. This may explain, at least partly, the known positive effect of light in somatic growth in Atlantic cod [22], [23]. The moderate to strong correlations found between the expression of muscle-related and clock genes could be linked to a possible circadian clock-regulated expression of muscle genes, which has been suggested in zebrafish skeletal muscle [21]. In mouse, approximately 7% of the muscle transcriptome displays circadian rhythmicity [6] and it is very likely a number of muscle-related genes under circadian control remain to be identified in fish.

## Conclusions

This is the first study discussing the possible relevance of a functional clock system in fast skeletal muscle of a teleost other than zebrafish. We reported that several clock genes are transcribed in fast skeletal muscle of Atlantic cod, suggesting the presence of a complex peripheral clock in this tissue (Fig. 8). It is plausible that muscle development and growth may be under clock control, as evidenced by the daily rhythmicity of *myf5* and *mbnl1* and strong correlation observed between transcript levels of muscle-related and clock genes. Taken together, our data provide a novel comparative perspective to our current knowledge about the implication of circadian clocks in muscle physiology and raises important questions. In particular, the autonomy of this peripheral clock and the extent of its importance in regulating the muscle transcriptome remain to be ascertained.

## Supporting Information

Figure S1 **Radiation trees of clock genes from Atlantic cod.** Genes were identified from the transcriptional activation arm (A: *arntl*; B: *clock*; C: *npas*), repression arm (D: *cry*; E: *per*; F: *tim*) and the stabilizing loop (G: *nr1d* H: *rora*). The unrooted trees were constructed by maximum likelihood using an LG substitution model with four substitution rate categories. Branch support was determined by aLRT SH-like tests. The Atlantic cod clock genes cloned in the present study are highlighted in red bold font. Teleostean clades are circled by a dotted red line.
(PDF)

Figure S2 **Partial synteny map of Atlantic cod clock genes (A:** ***arntl1*** **; B:** ***arntl2*** **; C:** ***clock*** **; D:** ***npas1*** **; E:** ***npas2*** **; F:** ***cry1*** **; G:** ***cry-dash*** **; H:** ***cry2*** **; I:** ***cry3*** **; J:** ***per1*** **; K:** ***per2*** **; L:** ***tim*** **; M:** ***nr1d1*** **; N:** ***nr1d2*** **; O:** ***rora*** **).** Orthologous genes in *G. morhua*, *D. rerio*; *O. latipes*, *T. rubripes*, *T. nigroviridis*, *O. niloticus* and *X. maculatus* are indicated by block arrows showing their position and orientation (un: unidentified gene).
(PDF)

Table S1 **Gene name, GenBank accession number, genomic location, primer sequences and thermocycling parameters of the identified Atlantic cod clock genes.**
(PDF)

Table S2 **List of primers for reference genes and muscle-related genes used in this study.**
(PDF)

Table S3 **Parameters defining the daily rhythmic expression of muscle-related genes.**
(PDF)

Table S4 **Correlation indices of clock and muscle-related gene expression.**
(PDF)

## References

[1] Vatine G, Vallone D, Gothilf Y, Foulkes NS (2011) It's time to swim! Zebrafish and the circadian clock. FEBS Lett 585: 1485–1494. 21486566 10.1016/j.febslet.2011.04.007

[2] Wang H (2008) Comparative analysis of period genes in teleost fish genomes. J Mol Evol 67: 29–40. 18535754 10.1007/s00239-008-9121-5

[3] Wang H (2008) Comparative analysis of teleost fish genomes reveals preservation of different ancient clock duplicates in different fishes. Mar Genomics 1: 69–78. 21798156 10.1016/j.margen.2008.06.003

[4] Sharma VK (2003) Adaptive Significance of Circadian Clocks. Chronobiol Int 20: 901–919. 14680135 10.1081/cbi-120026099

[5] Lowrey PL, Takahashi JS (2004) Mammalian circadian biology: Elucidating genome-wide levels of temporal organization. pp. 407–441. 10.1146/annurev.genom.5.061903.175925 PMC3770722 15485355

[6] McCarthy JJ, Andrews JL, McDearmon EL, Campbell KS, Barber BK, et al (2007) Identification of the circadian transcriptome in adult mouse skeletal muscle. Physiol Genomics 31: 86–95. 17550994 10.1152/physiolgenomics.00066.2007 PMC6080860

[7] Ptitsyn AA, Zvonic S, Conrad SA, Scott LK, Mynatt RL, et al (2006) Circadian Clocks Are Resounding in Peripheral Tissues. PLoS Comput Biol 2: e16. 16532060 10.1371/journal.pcbi.0020016 PMC1391918

[8] Lee C, Bae K, Edery I (1999) PER and TIM inhibit the DNA binding activity of a *Drosophila* CLOCK-CYC/dBMAL1 heterodimer without disrupting formation of the heterodimer: a basis for circadian transcription. Mol Cell Biol 19: 5316–5325. 10409723 10.1128/mcb.19.8.5316 PMC84375

[9] Cuesta IH, Lahiri K, Lopez-Olmeda JF, Loosli F, Foulkes NS, et al (2014) Differential maturation of rhythmic clock gene expression during early development in medaka (*Oryzias latipes*). Chronobiol Int 31: 468–478. 24456338 10.3109/07420528.2013.856316

[10] Sánchez JA, Madrid JA, Sánchez-Vázquez FJ (2010) Molecular cloning, tissue distribution, and daily rhythms of expression of per1 gene in European sea bass (*Dicentrarchus labrax*). Chronobiol Int 27: 19–33. 20205555 10.3109/07420520903398633

[11] Martín-Robles ÁJ, Whitmore D, Sánchez-Vázquez FJ, Pendón C, Muñoz-Cueto JA (2012) Cloning, tissue expression pattern and daily rhythms of Period1, Period2, and Clock transcripts in the flatfish Senegalese sole, *Solea senegalensis* . J Comp Physiol B 182: 673–685. 22373774 10.1007/s00360-012-0653-z

[12] Patiño MAL, Rodríguez-Illamola A, Conde-Sieira M, Soengas JL, Míguez JM (2011) Daily rhythmic expression patterns of *clock1a*, *bmal1*, and *per1* genes in retina and hypothalamus of the rainbow trout, *Oncorhynchus mykiss* . Chronobiol Int 28: 381–389. 21721853 10.3109/07420528.2011.566398

[13] Huang TS, Ruoff P, Fjelldal PG (2010) Effect of continuous light on daily levels of plasma melatonin and cortisol and expression of clock genes in pineal gland, brain, and liver in Atlantic salmon postsmolts. Chronobiol Int 27: 1715–1734. 20969519 10.3109/07420528.2010.521272

[14] Cavallari N, Frigato E, Vallone D, Fröhlich N, Lopez-Olmeda JF, et al (2011) A blind circadian clock in cavefish reveals that opsins mediate peripheral clock photoreception. PLoS Biol 9: e1001142. 21909239 10.1371/journal.pbio.1001142 PMC3167789

[15] Falcón J (1999) Cellular circadian clocks in the pineal. Prog Neurobiol 58: 121–162. 10338357 10.1016/s0301-0082(98)00078-1

[16] Dardente H, Cermakian N (2007) Molecular circadian rhythms in central and peripheral clocks in mammals. Chronobiol Int 24: 195–213. 17453843 10.1080/07420520701283693

[17] Richards J, Gumz ML (2012) Advances in understanding the peripheral circadian clocks. FASEB J 26: 3602–3613. 22661008 10.1096/fj.12-203554 PMC3425819

[18] Whitmore D, Foulkes NS, Sassone-Corsi P (2000) Light acts directly on organs and cells in culture to set the vertebrate circadian clock. Nature 404: 87–91. 10716448 10.1038/35003589

[19] Andrews JL, Zhang X, McCarthy JJ, McDearmon EL, Hornberger TA, et al (2010) CLOCK and BMAL1 regulate MyoD and are necessary for maintenance of skeletal muscle phenotype and function. Proc Natl Acad Sci U S A 107: 19090–19095. 20956306 10.1073/pnas.1014523107 PMC2973897

[20] Lefta M, Wolff G, Esser KA (2011) Chapter nine - Circadian Rhythms, the Molecular Clock, and Skeletal Muscle. In: Grace k P, editor. Current Topics in Developmental Biology: Academic Press. pp. 231–271. 10.1016/B978-0-12-385940-2.00009-7 PMC4545213 21621073

[21] Amaral IPG, Johnston IA (2012) Circadian expression of clock and putative clock-controlled genes in skeletal muscle of the zebrafish. Am J Physiol Regul Integr Comp Physiol 302: R193–R206. 22031781 10.1152/ajpregu.00367.2011

[22] Taranger GL, Aardal L, Hansen T, Kjesbu OS (2006) Continuous light delays sexual maturation and increases growth of Atlantic cod (*Gadus morhua* L.) in sea cages. ICES J Mar Sci 63: 365–375.

[23] Nagasawa K, Giannetto A, Fernandes JMO (2012) Photoperiod influences growth and MLL (mixed-lineage leukaemia) expression in Atlantic cod. PLoS ONE 7: e36908. 22590633 10.1371/journal.pone.0036908 PMC3348894

[24] Campos C, Valente LMP, Borges P, Bizuayehu T, Fernandes JMO (2010) Dietary lipid levels have a remarkable impact on the expression of growth-related genes in Senegalese sole (*Solea senegalensis* Kaup). J Exp Biol 213: 200–209. 20038653 10.1242/jeb.033126

[25] Bayarri MJ, Madrid JA, Sánchez-Vázquez FJ (2002) Influence of light intensity, spectrum and orientation on sea bass plasma and ocular melatonin. J Pineal Res 32: 34–40. 11841598 10.1034/j.1600-079x.2002.10806.x

[26] Ruangsri J, Salger SA, Caipang CMA, Kiron V, Fernandes JMO (2012) Differential expression and biological activity of two piscidin paralogues and a novel splice variant in Atlantic cod (*Gadus morhua* L.). Fish Shellfish Immunol 32: 396–406. 22178249 10.1016/j.fsi.2011.11.022

[27] Fernandes JMO, Mommens M, Hagen Ø, Babiak I, Solberg C (2008) Selection of suitable reference genes for real-time PCR studies of Atlantic halibut development. Comp Biochem Physiol B Biochem Mol Biol 150: 23–32. 18302990 10.1016/j.cbpb.2008.01.003

[28] Campos C, Valente LMP, Fernandes JMO (2012) Molecular evolution of zebrafish dnmt3 genes and thermal plasticity of their expression during embryonic development. Gene 500: 93–100. 22450363 10.1016/j.gene.2012.03.041

[29] Velarde E, Haque R, Iuvone PM, Azpeleta C, Alonso-Gomez AL, et al (2009) Circadian clock genes of goldfish, carassius auratus: CDNA cloning and rhythmic expression of period and cryptochrome transcripts in retina, liver, and gut. J Biol Rhythms 24: 104–113. 19346448 10.1177/0748730408329901 PMC2666933

[30] Woods IG, Wilson C, Friedlander B, Chang P, Reyes DK, et al (2005) The zebrafish gene map defines ancestral vertebrate chromosomes. Genome Res 15: 1307–1314. 16109975 10.1101/gr.4134305 PMC1199546

[31] Kobayashi K, Kanno SI, Smit B, Van Der Horst GTJ, Takao M, et al (1998) Characterization of photolyase/blue-light receptor homologs in mouse and human cells. Nucleic Acids Res 26: 5086–5092. 9801304 10.1093/nar/26.22.5086 PMC147960

[32] Hughes AL (2005) Gene duplication and the origin of novel proteins. Proc Natl Acad Sci U S A 102: 8791–8792. 15956198 10.1073/pnas.0503922102 PMC1157061

[33] Hughes AL (2002) Adaptive evolution after gene duplication. Trends Genet 18: 433–434. 12175796 10.1016/s0168-9525(02)02755-5

[34] Ohta T (1990) How gene families evolve. Theor Popul Biol 37: 213–219. 2326764 10.1016/0040-5809(90)90036-u

[35] Dekens MP, Whitmore D (2008) Autonomous onset of the circadian clock in the zebrafish embryo. Embo J 27: 2757–2765. 18800057 10.1038/emboj.2008.183 PMC2572168

[36] Weger M, Weger BD, Diotel N, Rastegar S, Hirota T, et al (2013) Real-time in vivo monitoring of circadian E-box enhancer activity: a robust and sensitive zebrafish reporter line for developmental, chemical and neural biology of the circadian clock. Dev Biol 380: 259–273. 23665472 10.1016/j.ydbio.2013.04.035

[37] Busza A, Emery-Le M, Rosbash M, Emery P (2004) Roles of the two *Drosophila* cryptochrome structural domains in circadian photoreception. Science 304: 1503–1506. 15178801 10.1126/science.1096973

[38] López-Olmeda JF, Tartaglione EV, De La Iglesia HO, Sánchez-Vázquez FJ (2010) Feeding entrainment of food-anticipatory activity and per1 expression in the brain and liver of zebrafish under different lighting and feeding conditions. Chronobiol Int 27: 1380–1400. 20795882 10.3109/07420528.2010.501926

[39] Migaud H, Davie A, Taylor JF (2010) Current knowledge on the photoneuroendocrine regulation of reproduction in temperate fish species. J Fish Biol 76: 27–68. 20738699 10.1111/j.1095-8649.2009.02500.x

[40] Ángeles Esteban M, Cuesta A, Rodríguez A, Meseguer J (2006) Effect of photoperiod on the fish innate immune system: A link between fish pineal gland and the immune system. J Pineal Res 41: 261–266. 16948787 10.1111/j.1600-079X.2006.00362.x

[41] Bowden TJ, Thompson KD, Morgan AL, Gratacap RML, Nikoskelainen S (2007) Seasonal variation and the immune response: A fish perspective. Fish Shellfish Immunol 22: 695–706. 17116408 10.1016/j.fsi.2006.08.016

[42] Giannetto A, Fernandes JMO, Nagasawa K, Mauceri A, Maisano M, et al (2014) Influence of continuous light treatment on expression of stress biomarkers in Atlantic cod. Dev Comp Immunol 44: 30–34. 24296437 10.1016/j.dci.2013.11.011

[43] Guillaumond F, Dardente H, Giguère V, Cermakian N (2005) Differential control of Bmal1 circadian transcription by REV-ERB and ROR nuclear receptors. J Biol Rhythms 20: 391–403. 16267379 10.1177/0748730405277232

[44] Cermakian N, Whitmore D, Foulkes NS, Sassone-Corsi P (2000) Asynchronous oscillations of two zebrafish CLOCK partners reveal differential clock control and function. Proc Natl Acad Sci U S A 97: 4339–4344. 10760301 10.1073/pnas.97.8.4339 PMC18243

[45] Hogenesch JB, Gu YZ, Jain S, Bradfield CA (1998) The basic-helix-loop-helix-PAS orphan MOP3 forms transcriptionally active complexes with circadian and hypoxia factors. Proc Natl Acad Sci U S A 95: 5474–5479. 9576906 10.1073/pnas.95.10.5474 PMC20401

[46] Takahata S, Sogawa K, Kobayashi A, Ema M, Mimura J, et al (1998) Transcriptionally active heterodimer formation of an Arnt-like PAS protein, Arnt3, with HIF-1a, HLF, and clock. Biochem Biophys Res Commun 248: 789–794. 9704006 10.1006/bbrc.1998.9012

[47] del Pozo A, Vera LM, Sánchez JA, Sánchez-Vázquez FJ (2012) Molecular cloning, tissue distribution and daily expression of *cry1* and *cry2* clock genes in European seabass (*Dicentrarchus labrax*). Comp Biochem Physiol A Mol Integr Physiol 163: 364–371. 22841604 10.1016/j.cbpa.2012.07.004

[48] Weger BD, Sahinbas M, Otto GW, Mracek P, Armant O, et al (2011) The light responsive transcriptome of the zebrafish: function and regulation. PLoS ONE 6: e17080. 21390203 10.1371/journal.pone.0017080 PMC3039656

[49] Reppert SM, Weaver DR (2002) Coordination of circadian timing in mammals. Nature 418: 935–941. 12198538 10.1038/nature00965

[50] Crumbley C, Wang Y, Kojetin DJ, Burris TP (2010) Characterization of the core mammalian clock component, NPAS2, as a REV-ERBα/RORα target gene. J Biol Chem 285: 35386–35392. 20817722 10.1074/jbc.M110.129288 PMC2975162

[51] Crumbley C, Burris TP (2011) Direct regulation of CLOCK expression by REV-ERB. PLoS ONE 6: e17290. 21479263 10.1371/journal.pone.0017290 PMC3066191

