# Supplementary material for: Daily Rhythmicity of Clock Gene Transcripts in Atlantic Cod Fast Skeletal Muscle
Source: PLoS One. 2014 Jun 12;9(6):e99172. doi: 10.1371/journal.pone.0099172 (PMC4062345; doi:10.1371/journal.pone.0099172)

Figure S1

A. Arntl

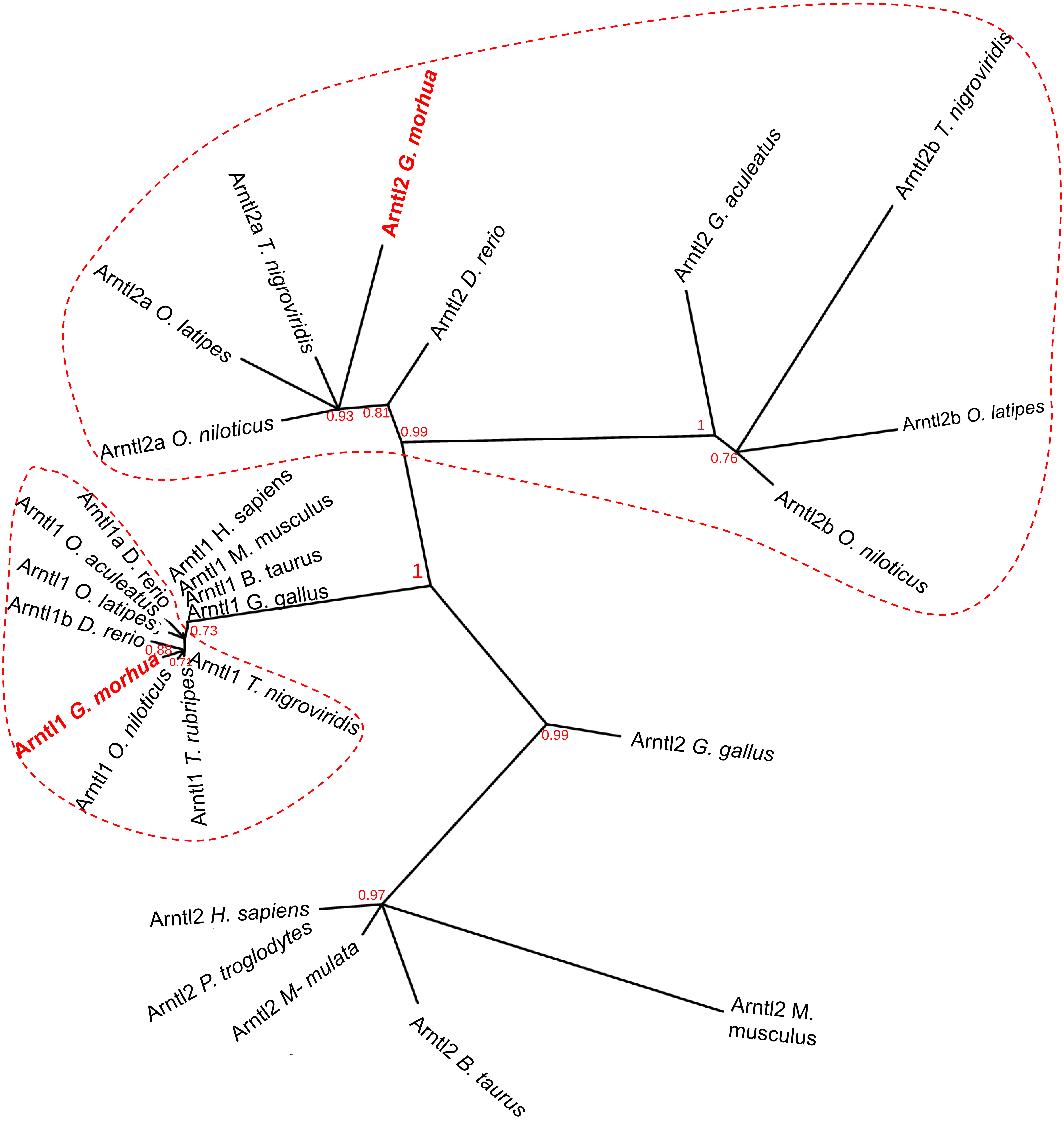

Figure S1

B. Clock

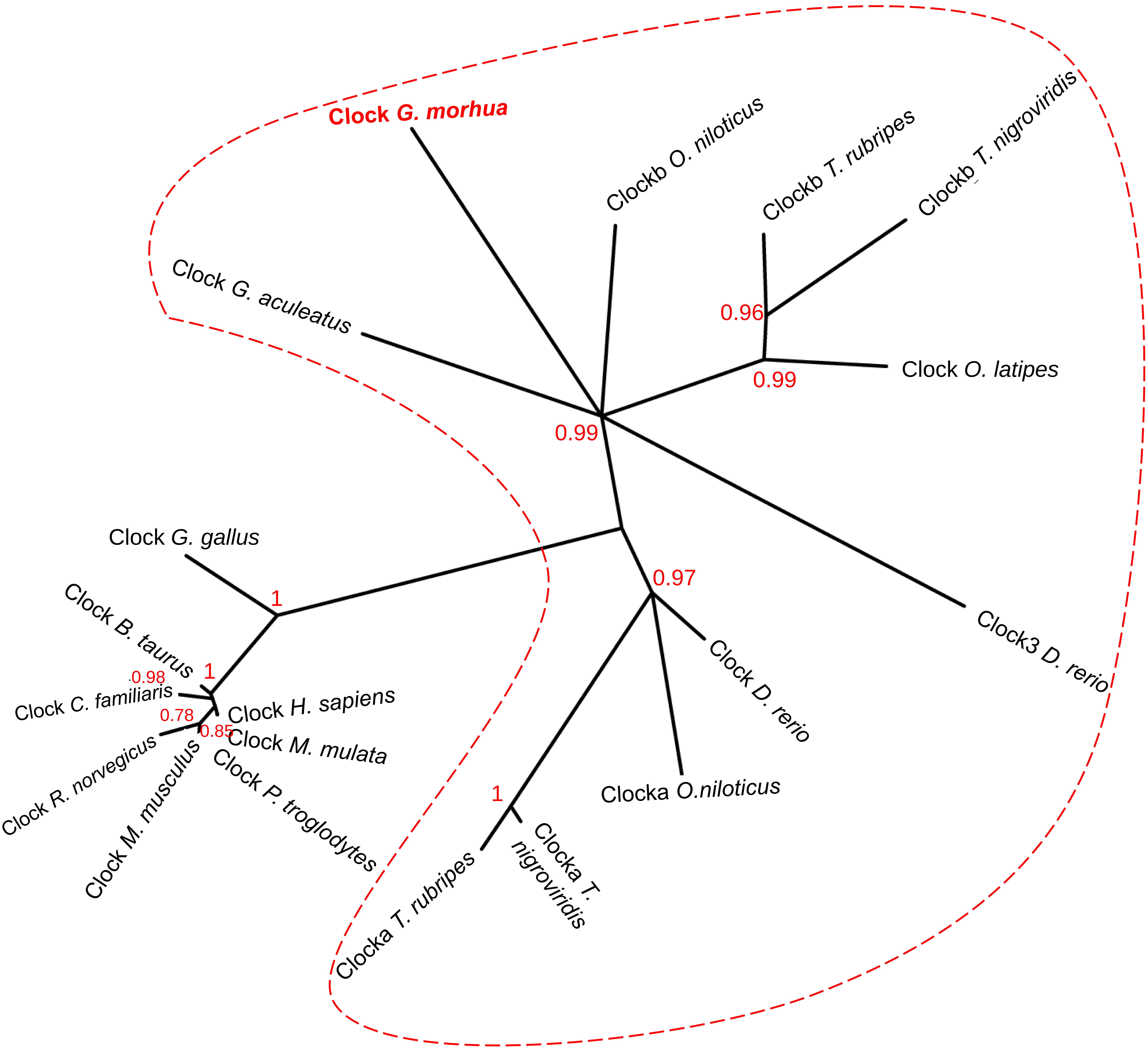

Figure S1

C. Npas

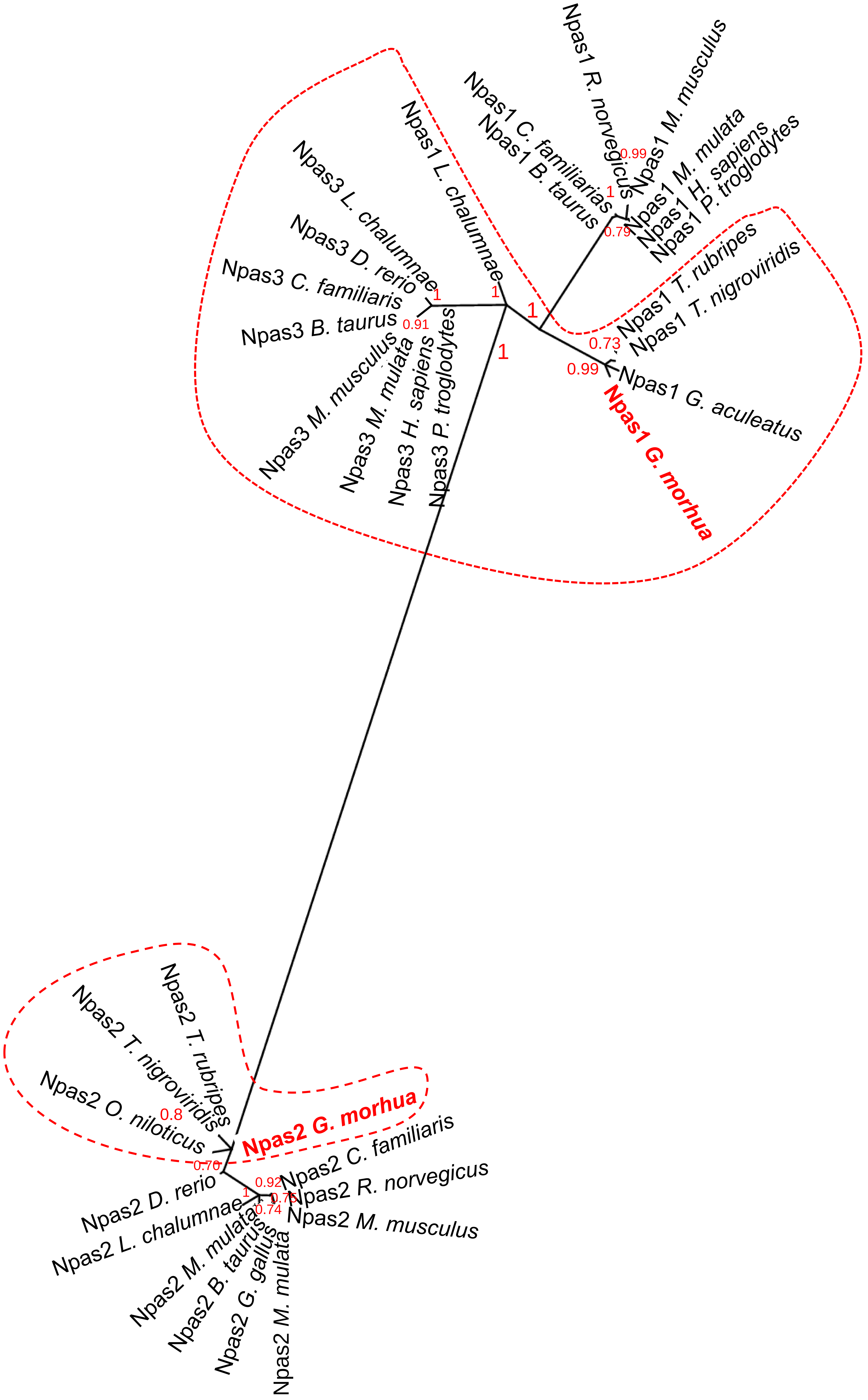

Figure S1

D. Cry

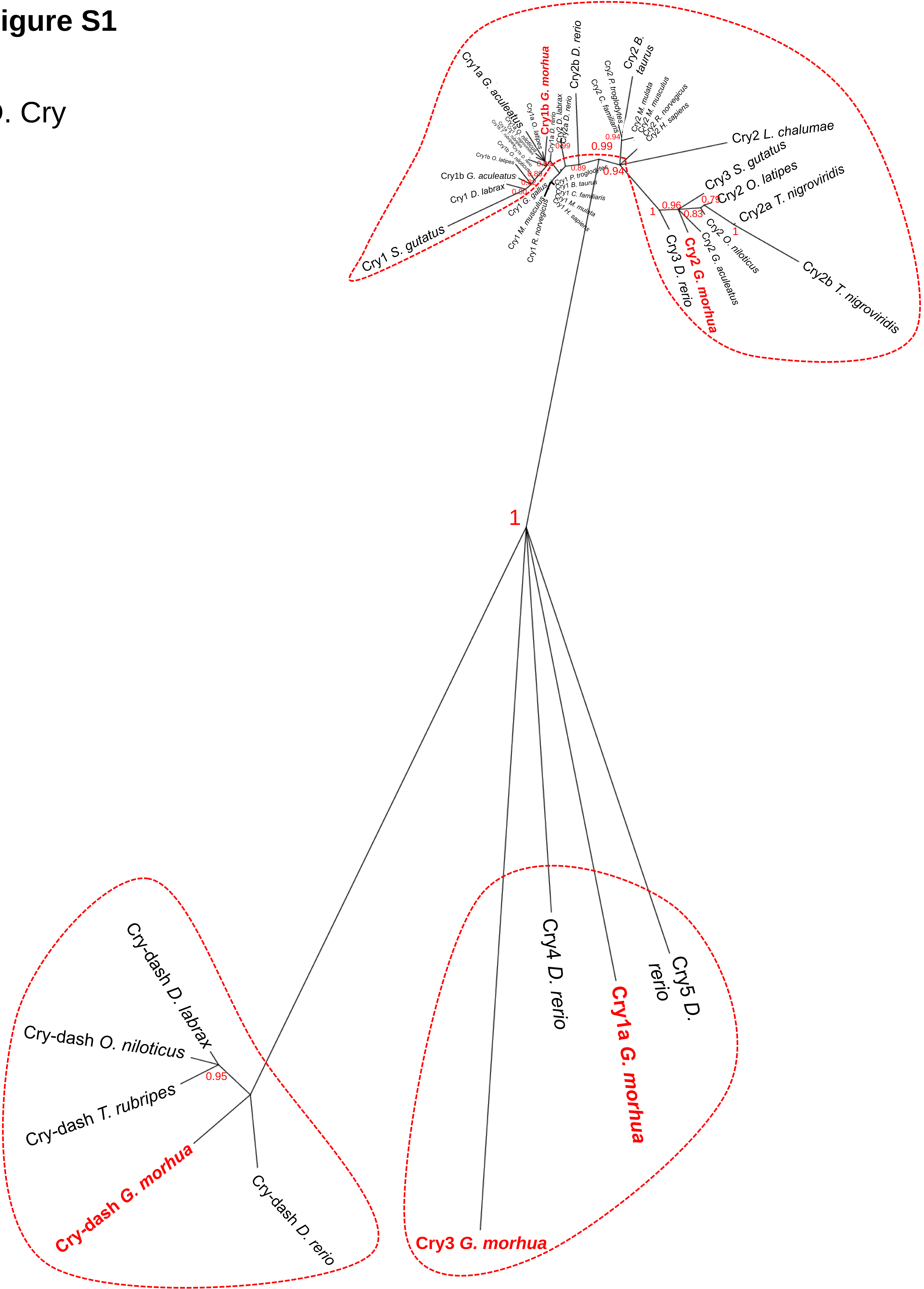

Figure S1

E. Per

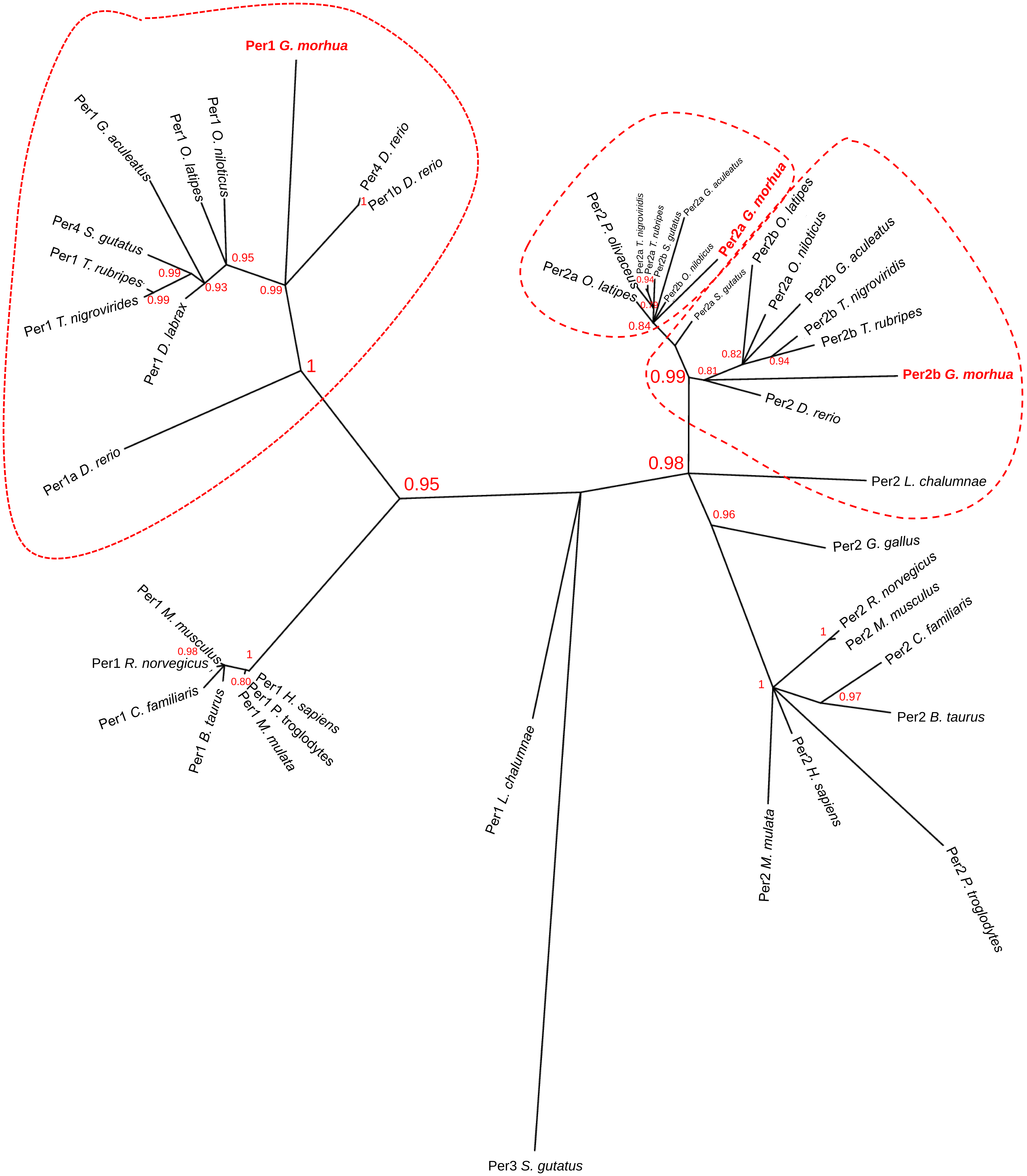

Figure S1

F. Tim

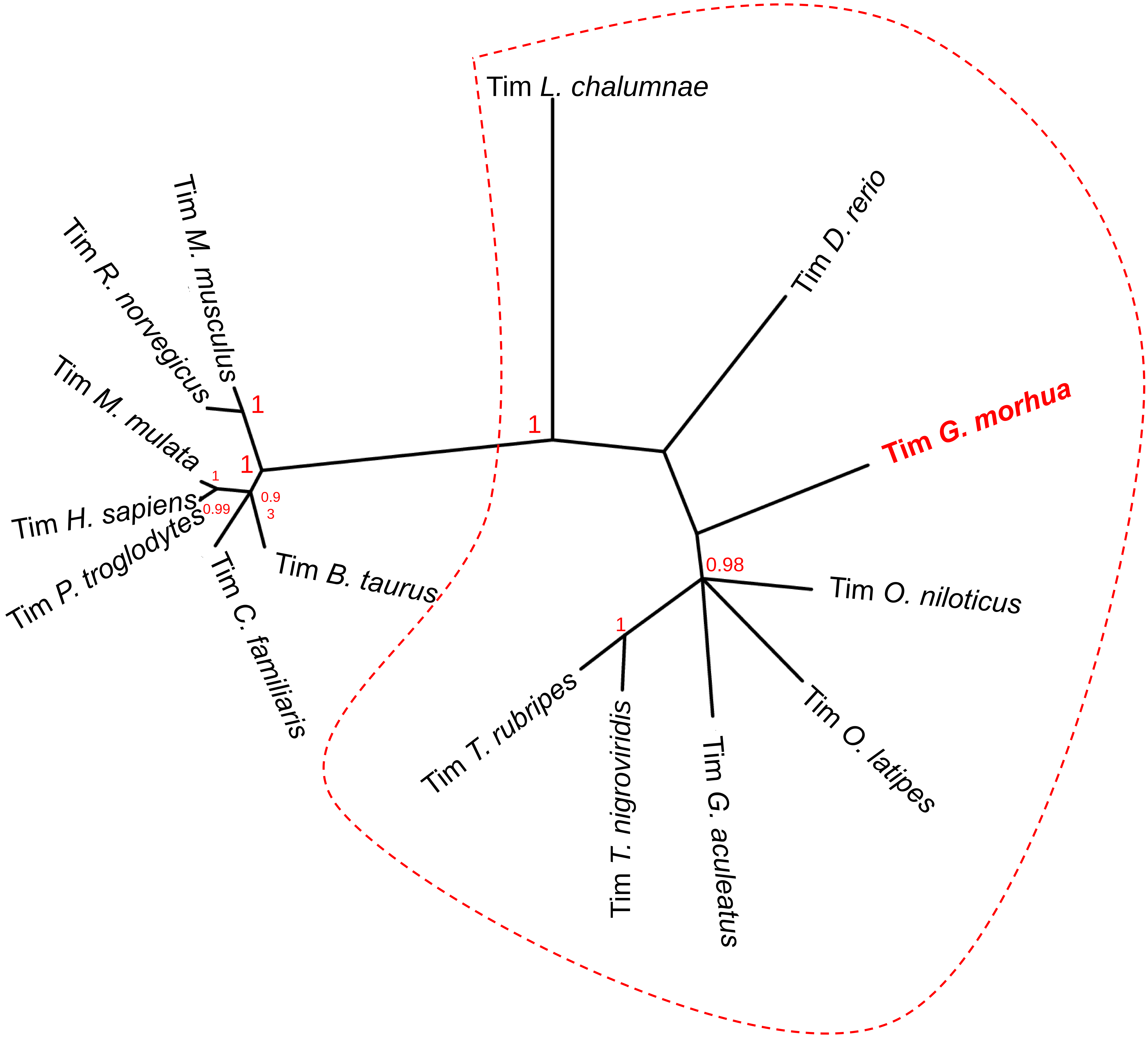

Figure S1

G. Nr1d

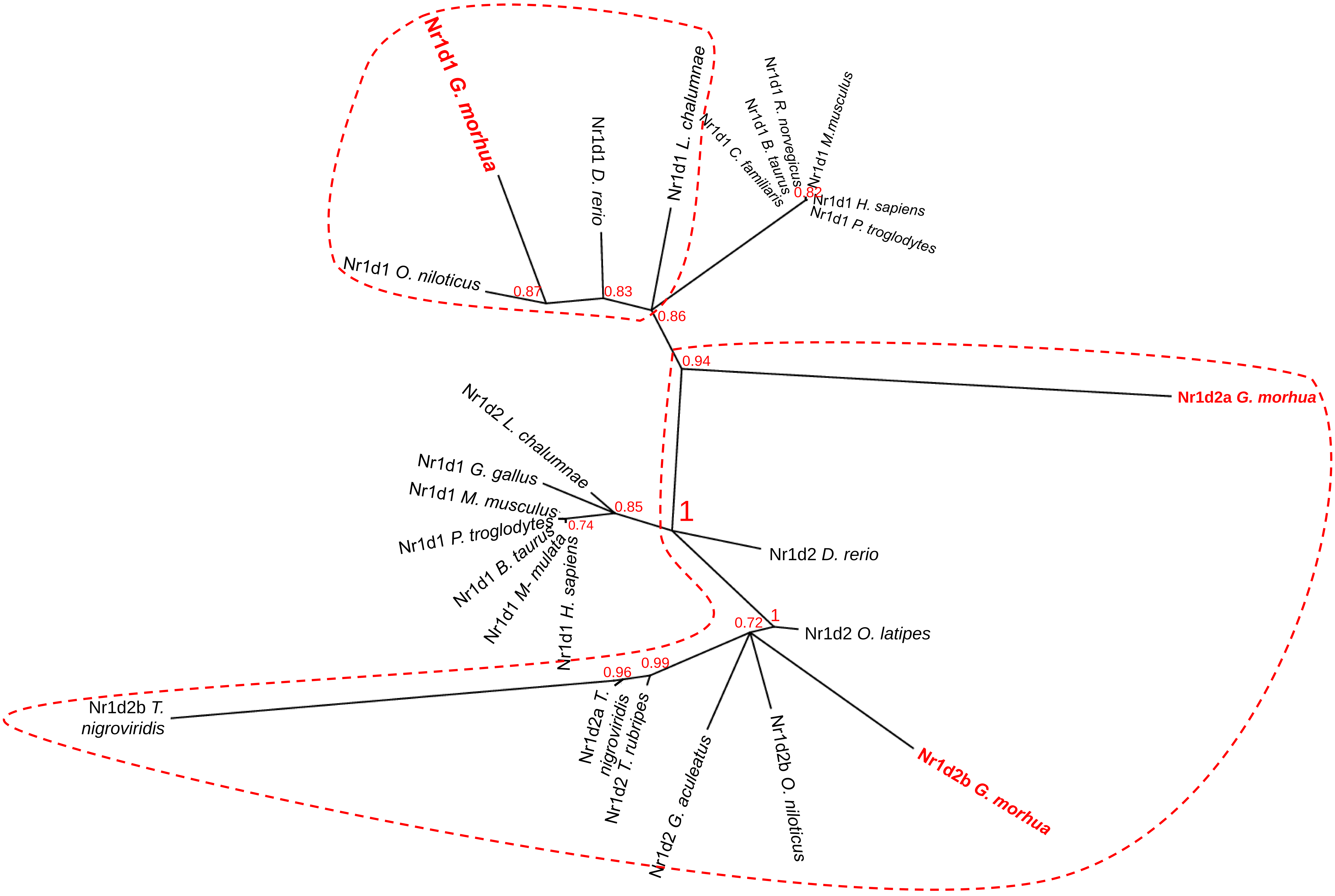

Figure S1

H. Rora

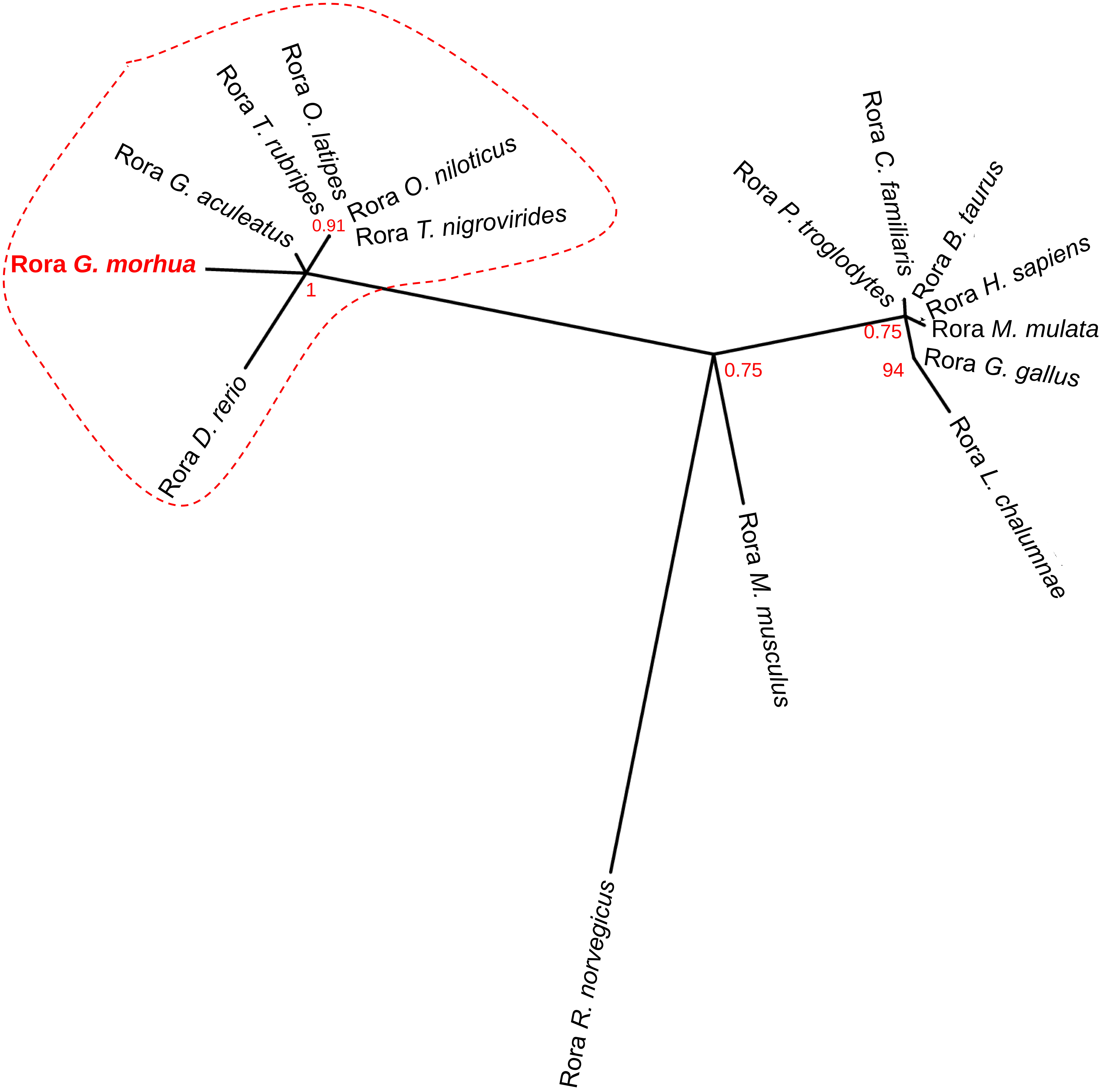

Supplement: Figure S1 — Radiation trees of clock genes from Atlantic cod. Genes were identified from the transcriptional activation arm (A: arntl; B: clock; C: npas), repression arm (D: cry; E: per; F: tim) and the stabilizing loop (G: nr1d H: rora). The unrooted trees were constructed by maximum likelihood using an LG substitution model with four substitution rate categories. Branch support was determined by aLRT SH-like tests. The Atlantic cod clock genes cloned in the present study are highlighted in red bold font. Teleostean clades are circled by a dotted red line. (PDF) [file pone.0099172.s001.pdf]
