# Supplementary material for: Daily Rhythmicity of Clock Gene Transcripts in Atlantic Cod Fast Skeletal Muscle
Source: PLoS One. 2014 Jun 12;9(6):e99172. doi: 10.1371/journal.pone.0099172 (PMC4062345; doi:10.1371/journal.pone.0099172)
Supplement: Figure S2 — Partial synteny map of Atlantic cod clock genes (A: arntl1 ; B: arntl2 ; C: clock ; D: npas1 ; E: npas2 ; F: cry1 ; G: cry-dash ; H: cry2 ; I: cry3 ; J: per1 ; K: per2 ; L: tim ; M: nr1d1 ; N: nr1d2 ; O: rora ). Orthologous genes in G. morhua, D. rerio; O. latipes, T. rubripes, T. nigroviridis, O. niloticus and X. maculatus are indicated by block arrows showing their position and orientation (un: unidentified gene). (PDF) [file pone.0099172.s002.pdf]

Figure S2

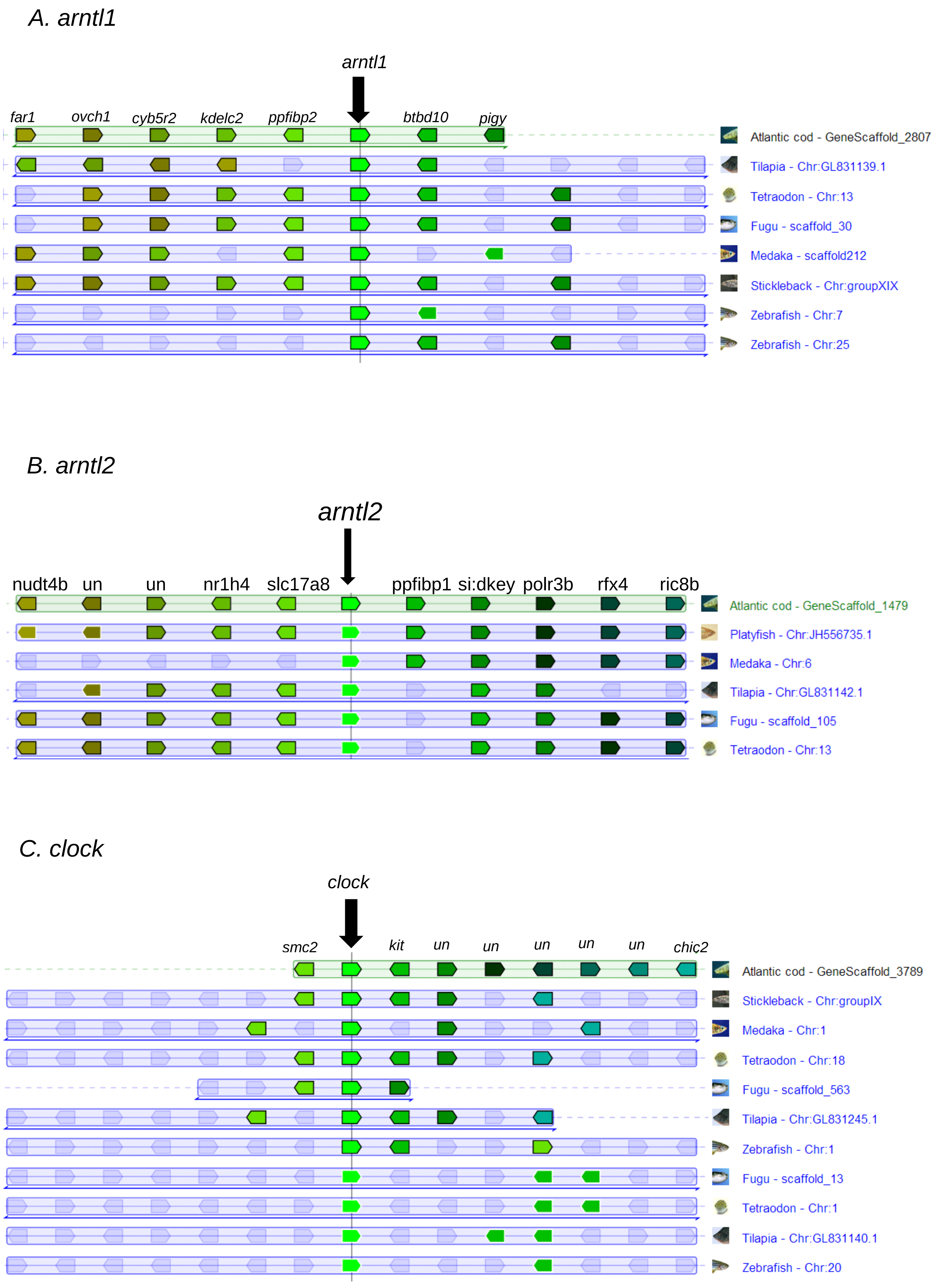
A. arntl1
arntl1
far1
ovch1
cyb5r2
kdelc2
ppfibp2
btbd10
pigy
Atlantic cod - GeneScaffold_2807
Tilapia - Chr:GL831139.1
Tetraodon - Chr:13
Fugu - scaffold_30
Medaka - scaffold212
Stickleback - Chr:groupXIX
Zebrafish - Chr:7
Zebrafish - Chr:25
B. arntl2
arntl2
nudt4b
un
un
nr1h4
slc17a8
ppfibp1
si:dkey
polr3b
rfx4
ric8b
Atlantic cod - GeneScaffold_1479
Platyfish - Chr:JH556735.1
Medaka - Chr:6
Tilapia - Chr:GL831142.1
Fugu - scaffold_105
Tetraodon - Chr:13
C. clock
clock
smc2
kit
un
un
un
un
un
chic2
Atlantic cod - GeneScaffold_3789
Stickleback - Chr:groupIX
Medaka - Chr:1
Tetraodon - Chr:18
Fugu - scaffold_563
Tilapia - Chr:GL831245.1
Zebrafish - Chr:1
Fugu - scaffold_13
Tetraodon - Chr:1
Tilapia - Chr:GL831140.1
Zebrafish - Chr:20

Figure S2

D. *npas1*

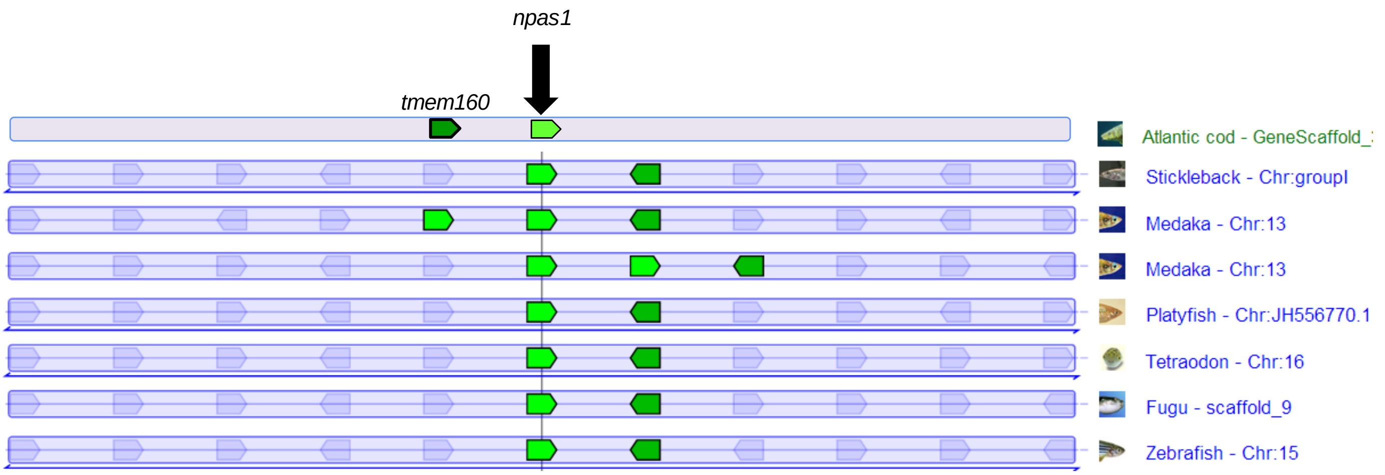

E. *npas2*

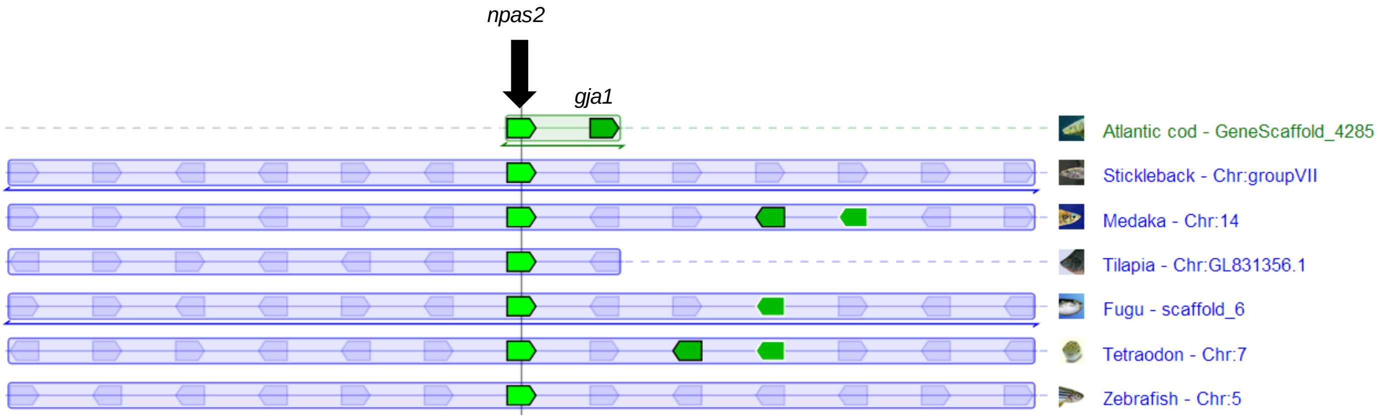

F. *cry1*

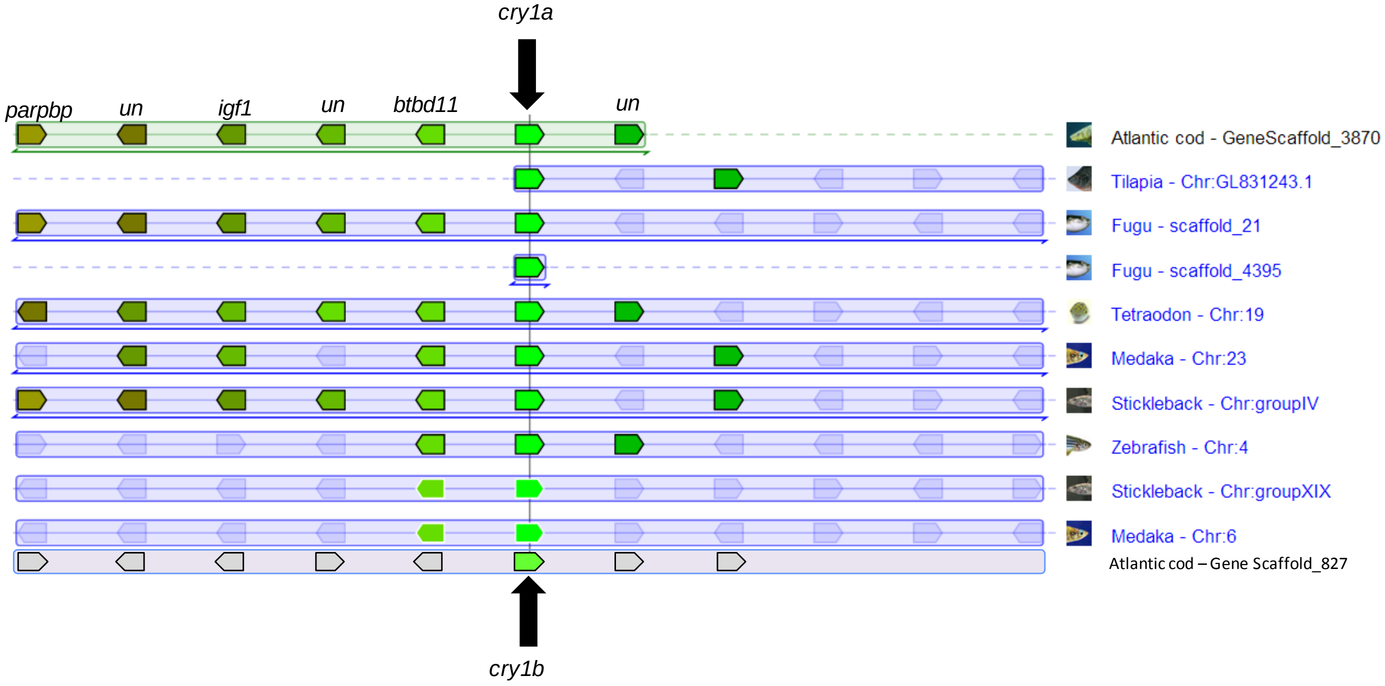

Figure S2

G. *cry-dash*

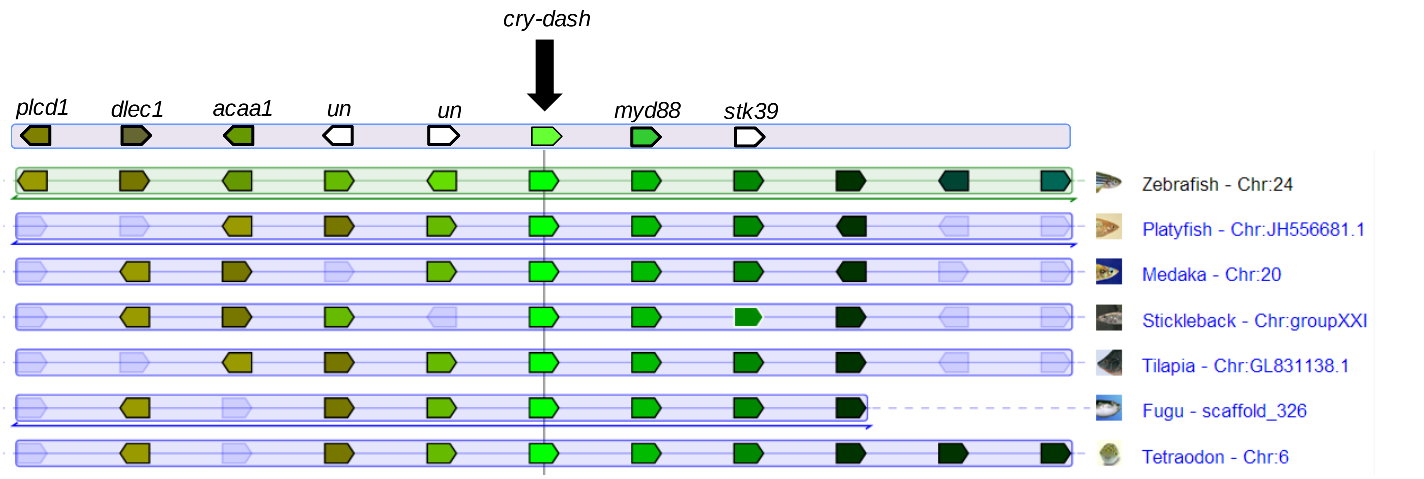

H. *cry2*

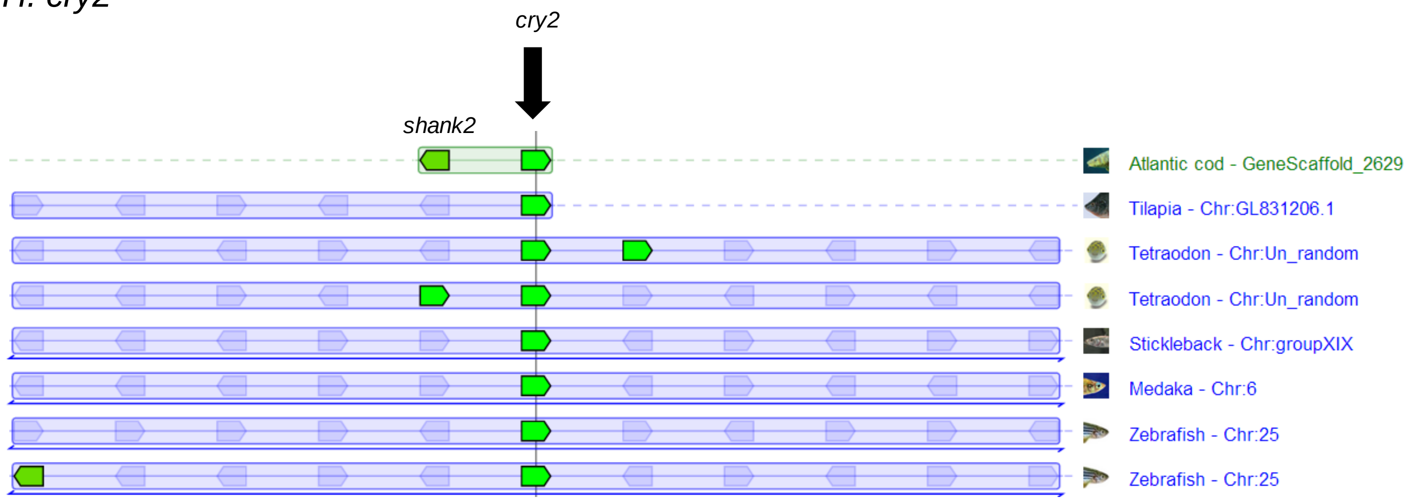

I. *cry3*

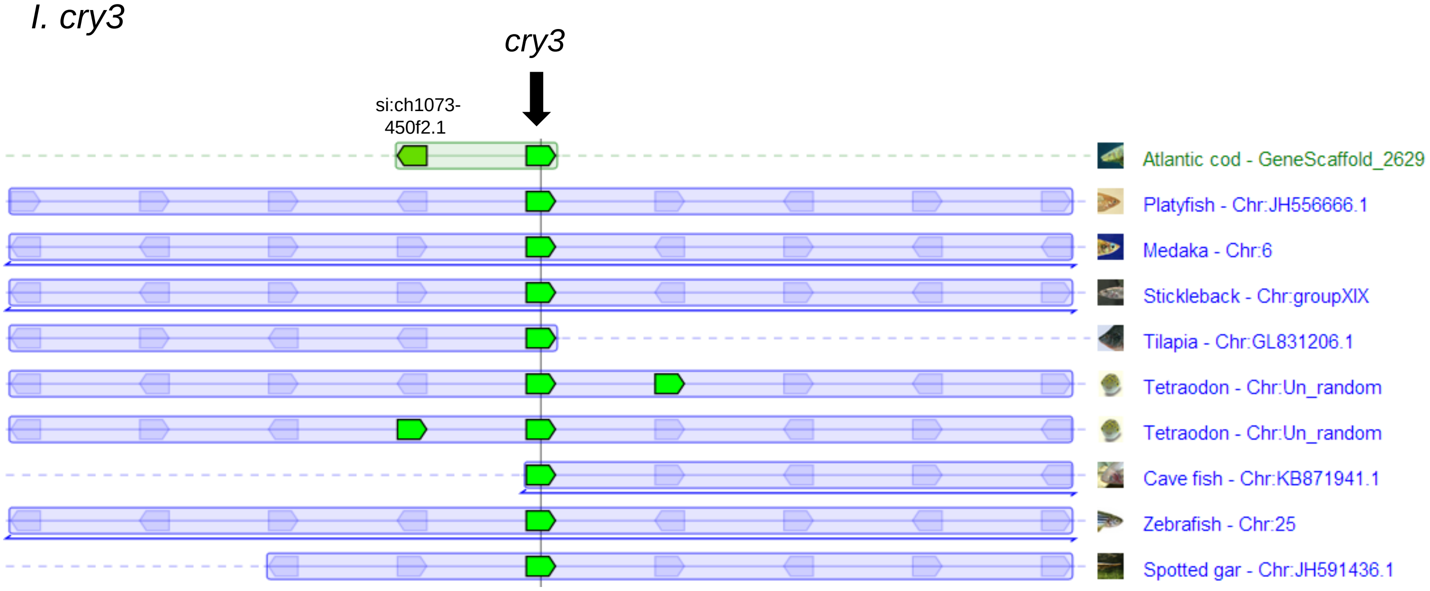

Figure S2

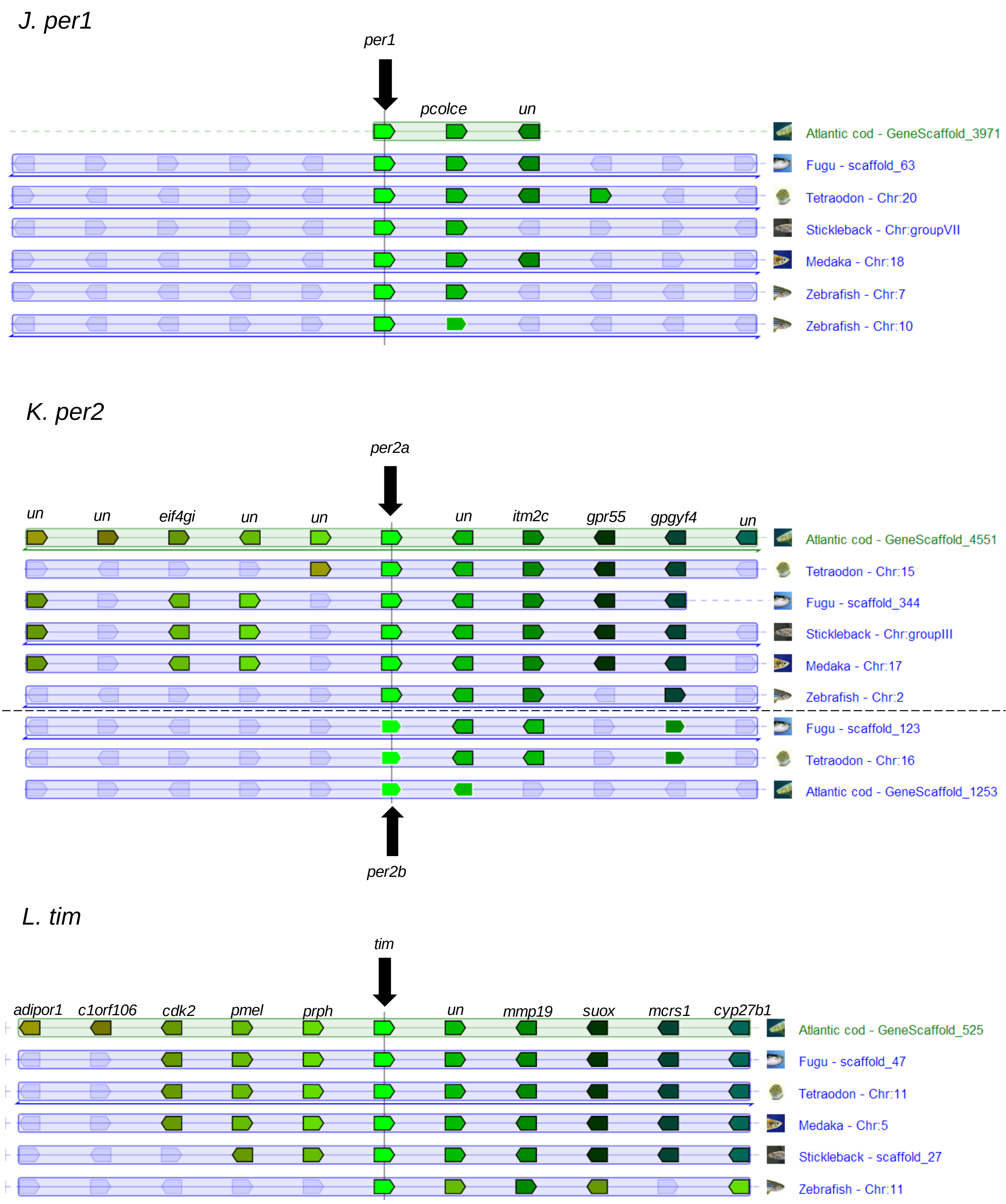

J. per1
per1
pcolce
un
Atlantic cod - GeneScaffold_3971
Fugu - scaffold_63
Tetraodon - Chr:20
Stickleback - Chr:groupVII
Medaka - Chr:18
Zebrafish - Chr:7
Zebrafish - Chr:10
K. per2
per2a
un
un
eif4gi
un
un
un
itm2c
gpr55
gpgyf4
un
Atlantic cod - GeneScaffold_4551
Tetraodon - Chr:15
Fugu - scaffold_344
Stickleback - Chr:groupIII
Medaka - Chr:17
Zebrafish - Chr:2
Fugu - scaffold_123
Tetraodon - Chr:16
Atlantic cod - GeneScaffold_1253
per2b
L. tim
tim
adipor1
c1orf106
cdk2
pmel
prph
un
mmp19
suox
mcrs1
cyp27b1
Atlantic cod - GeneScaffold_525
Fugu - scaffold_47
Tetraodon - Chr:11
Medaka - Chr:5
Stickleback - scaffold_27
Zebrafish - Chr:11

Figure S2

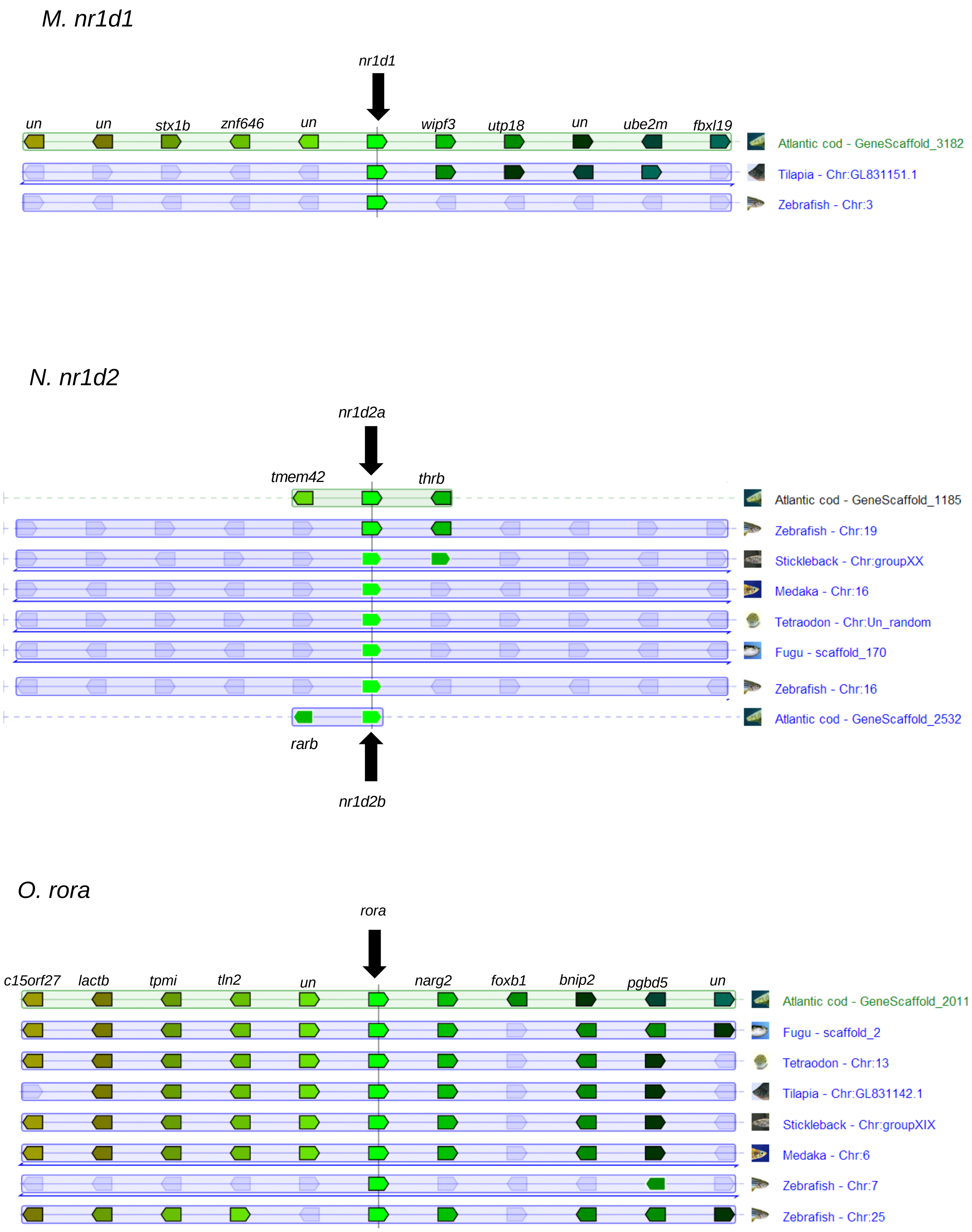
M. nr1d1
nr1d1
un
un
stx1b
znf646
un
wipf3
utp18
un
ube2m
fbxl19
Atlantic cod - GeneScaffold_3182
Tilapia - Chr:GL831151.1
Zebrafish - Chr:3
N. nr1d2
nr1d2a
tmem42
thrb
Atlantic cod - GeneScaffold_1185
Zebrafish - Chr:19
Stickleback - Chr:groupXX
Medaka - Chr:16
Tetraodon - Chr:Un_random
Fugu - scaffold_170
Zebrafish - Chr:16
Atlantic cod - GeneScaffold_2532
rarb
nr1d2b
O. rora
rora
c15orf27
lactb
tpmi
tln2
un
narg2
foxb1
bnip2
pgbd5
un
Atlantic cod - GeneScaffold_2011
Fugu - scaffold_2
Tetraodon - Chr:13
Tilapia - Chr:GL831142.1
Stickleback - Chr:groupXIX
Medaka - Chr:6
Zebrafish - Chr:7
Zebrafish - Chr:25
