# Supplementary material for: Daily Rhythmicity of Clock Gene Transcripts in Atlantic Cod Fast Skeletal Muscle
Source: PLoS One. 2014 Jun 12;9(6):e99172. doi: 10.1371/journal.pone.0099172 (PMC4062345; doi:10.1371/journal.pone.0099172)
Supplement: Table S1 — Gene name, GenBank accession number, genomic location, primer sequences and thermocycling parameters of the identified Atlantic cod clock genes. (PDF) [file pone.0099172.s003.pdf]

Table S1. Gene name, GenBank accession number, genomic location, primer sequences and thermocycling parameters of the identified Atlantic cod clock genes.

Gene name	GenBank Accession Number	Genomic location	Primer sequences	Annealing Temperature (^{o}C)	E	Amplicon size (bp)
[1]*arnt11*	JN643704	GeneScaffold_2807	F: AGGATGAAGTGCAACAGGCC<br>R: GATGGTGCAGAAGCTCTTGC	60	97	99
[1]*arntl2*	JX03864	GeneScaffold_1479	F: TCTCAAAGGTTCCTCTTCTTCGT<br>R: CACAAACAGAAAGCCATCGG	64	85	103
clock	JN643707	GeneScaffold_3789	F: CTGGAGCACCTACCGGCGGA<br>R: GCGGCGTGCGTCGACAGAGC	60	87	101
npas1	KC204820	GeneScaffold_1829	F: TACTGTGAGAACAGGATCTCGG<br>R: TGGCTCTGTCTCAGGTTGTC	66	na	113
npas2	JX035866	GeneScaffold_4285	F: CAACAATGTGCCTACGTCCTC<br>R: GAACAGGAACTTCCACTCCAG	65	104	199
cry1a	JN643708	GeneScaffold_3816	F: AACTTCGACCGCATGCTGG<br>R: GGTGGTGGATCCAGCCCTC	60	84	154
cry1b	KC204818	GeneScaffold_3870	F: GACCTCGACAGGATCGTAGAG<br>R: CTGCACTTCCAGGTAGAGCT	66	91	130
cry-dash	KC204819	GeneScaffold_827	F: GCCAATCAGAGCACCTACAGG<br>R: CCACGGAACAGACTTGTCTGG	66	115	126
cry2	JN643709	GeneScaffold_2629	F: GGCTGGATCTGCCCCGACG<br>R: CCCAGCTCTTCCAGTGAGGG	60	79	121
cry3	JX035865	GeneScaffold_2425	F: CGTACAAGGCTCTCAGGTGG<br>R: CAGGGCAGATGCAGGTAAG	60	95	176
per1	JN643710	GeneScaffold_3971	F: TTCGGCAGCATCGACTCGTCGG<br>R: CCTCCATGTCCCTGGAGGGC	60	82	178
per2a	JN643711	GeneScaffold_1253	F: GCTCCCATGATGTTCAACGCT<br>R: CCTCGATGATCTCAGATTCGG	64	101	186
per2b	JX035863	GeneScaffold_4551	F: CTGGGAGCGTCGTAGCCG<br>R: CGTATCAGCGGGGGTAAG	63	101	158
tim	JN634817	GeneScaffold_525	F: CTGGGGTACCTGGAGGGG<br>R: CACCATGAGCCTTGATGCA	65	104	213
rora	KC204821	GeneScaffold_2011	F: GTGTCAGAACCGATCAGATAGTG<br>R: AGTCGTCACAGCCTAATGAC	65	na	155
[2]*nr1d1*	KC204822	GeneScaffold_3182	F: GACTTCTCCCTCTCCTTCAG<br>R: CACCATTAGCACCTCAAAGG	60	77	135
[3]*nr1d2a*	JN643712	GeneScaffold_1771	F: CTGCAAGGGCTTCTTCCGTC<br>R: ATGTCCATGGTGGCGGACTC	63	79	152
nr1d2b	KC204823	GeneScaffold_2532	F:ACTTCTCCATGAGCTTTACTCC<br>R: AACCATTAGCACCTCGAAGG	64	119	134

na = not used for qPCR analysis
[1]*arntl1/2 is also known as brain and muscle Arnt-like 1/2, bmal1/2*
[2]*nr1d1 is also known as rev-erb alpha*
[3]*nr1d2 is also known as rev-erb beta*
