# Supplementary material for: Daily Rhythmicity of Clock Gene Transcripts in Atlantic Cod Fast Skeletal Muscle
Source: PLoS One. 2014 Jun 12;9(6):e99172. doi: 10.1371/journal.pone.0099172 (PMC4062345; doi:10.1371/journal.pone.0099172)
Supplement: Table S2 — List of primers for reference genes and muscle-related genes used in this study. (PDF) [file pone.0099172.s004.pdf]

Table S2. List of primers for reference genes and muscle-related genes used in this study.

Gene name	GenBank Accession Number	Primer sequences	Annealing Temperature (°C)	E	Amplicon size (bp)	Reference
arp	EX741373	F:TGATCCTCCACGACGATGAG<br>R:CAGGGCCTTGGCGAAGA	60	98	113	Olsvik et al..2008
eef1a	EX721840	F: CACTGCGGTGAAGTCCGTTG<br>R: GGGGTCGTTCTTGCTGTCT	60	93	79	Lilleeng et al.. 2007
ubi	EX735613	F: GGCCGCAAAGATGCAGAT<br>R: CTGGGCTCGACCTCAAGAGT	60	98	69	Olsvik et al.. 2008
luc	M15077	F: TCATTCTTCGCCAAAAGCACTCTG<br>R: AGCCCATATCCTTGTCGTATCCC	60	na	198	Campos et al.. 2012
myoD	AF329903	F: GGCATGATGGATTTCAACGG<br>R: CTCGAGAGGCACTCCAGGCT	60	89	128	This study
myoG	JQ582407	F: AACTTCGACCGCATGCTGG<br>R: GGTGGTGGATCCAGCCCTC	60	78	121	Nagasawa et al.. 2012
myf5	JQ619514	F: GACCTCGACAGGATCGTAGAG<br>R: CTGCACTTCCAGGTAGAGCT	66	78	140	Nagasawa et al.. 2012
myf6	KC204827	F: GCAGCGCCATCAGCTACATC<br>R: GGTCTGCAGAGGTCTGCAACTTC	66	77	165	This study
myhc	AY093703	F: CAGAAGCTATAAAAGGTGTCCG<br>R: GCAGCCATTCTTCTTATCCTCCTC	60	79	86	Koedijk et al.. 2010
pcna	KC204826	F: CCTCAGCAGTATGTCAAAGATCC<br>R: GATGGTTTCAAACACAAGAACGAG	60	74	105	This study
mstn	AF500271	F: GGCCACAGAGCCTGACCCCA<br>R: TCCGTCCGTAACGGGCGTCAG	60	126	184	This study
foxk2	KC204825	F: CAACTCCTGTCCGTCAAGTC<br>R: CTTTGAGTCATCCTTGGGACTG	64	75	148	This study
mbnl	KC204824	F: AATATTGTGCATGGGACCTG<br>R: TATCGGAATCTGATTGGCTGAG	63	90	126	This study

na = not used for qPCR analysis

Olsvik PA. Søfteland L. Lie KK (2008) Selection of reference genes for qRT-PCR examination of wild populations of Atlantic cod *Gadus morhua*. BMC Research Notes 1.

Lilleeng E. Frøystad MK. Vekterud K. Valen EC. Krogdahl Å (2007) Comparison of intestinal gene expression in Atlantic cod (*Gadus morhua*) fed standard fish meal or soybean meal by means of suppression subtractive hybridization and real-time PCR. Aquaculture 267: 269-283.

Campos C. Valente LMP. Fernandes JMO (2012) Molecular evolution of zebrafish dnmt3 genes and thermal plasticity of their expression during embryonic development. Gene 500: 93-100.

Nagasawa K. Giannetto A. Fernandes JMO (2012) Photoperiod influences growth and MLL (mixed-lineage leukaemia) expression in Atlantic cod. PLoS ONE 7.

Koedijk RM. Le François NR. Blier PU. Foss A. Folkvord A. et al. (2010) Ontogenetic effects of diet during early development on growth performance. myosin mRNA expression and metabolic enzyme activity in Atlantic cod juveniles reared at different salinities. Comparative Biochemistry and Physiology - A Molecular and Integrative Physiology 156: 102-109.
