# Supplementary material for: Daily Rhythmicity of Clock Gene Transcripts in Atlantic Cod Fast Skeletal Muscle
Source: PLoS One. 2014 Jun 12;9(6):e99172. doi: 10.1371/journal.pone.0099172 (PMC4062345; doi:10.1371/journal.pone.0099172)
Supplement: Table S3 — Parameters defining the daily rhythmic expression of muscle-related genes. (PDF) [file pone.0099172.s005.pdf]

Table S3. Parameters defining the daily rhythmic expression of muscle-related genes.

Gene name	Period	Mesor	Amplitude	Acrophase (h)	P value
myoD	24	0.21	0.068	3.58	0.49
<u>***myf5***</u>	24	0.35	0.089	7.49	0.15
mhyc	24	0.34	0.061	8.26	0.60
myoG	24	0.20	0.070	2.28	0.46
pcna	24	0.46	0.071	2.30	0.45
myf6	24	0.27	0.021	11.1	0.96
mstn	24	0.33	0.048	13.3	0.52
<u>***mbnl1***</u>	24	0.54	0.271	7.20	0.05
foxk2	24	0.32	0.048	6.36	0.69

NOTE: genes that are in bold font and underlined displayed daily rhythmicity in their expression.
