# Supplementary material for: Daily Rhythmicity of Clock Gene Transcripts in Atlantic Cod Fast Skeletal Muscle
Source: PLoS One. 2014 Jun 12;9(6):e99172. doi: 10.1371/journal.pone.0099172 (PMC4062345; doi:10.1371/journal.pone.0099172)
Supplement: Table S4 — Correlation indices of clock and muscle-related gene expression. (PDF) [file pone.0099172.s006.pdf]

Table S4. Correlation indices of clock and muscle-related gene expression.

**Muscle-related genes**	**Clock genes**															
	arntl1	*arntl2*	*clock*	*npas2*	*cry1a*	*cry1b*	*cry-dash*	*cry2*	*cry3*	*per1*	*per2a*	*per2b*	*tim*	*nr1d1a*	*nr1d2a*	*nr1d2b*
myoD	-0.65	-0.21	0.05	0.00	0.29	-0.12	0.45	0.52	0.02	-0.14	-0.39	-0.32	-0.02	0.60	0.06	0.04
myf5	0.09	-0.10	0.84	0.45	0.45	-0.13	0.26	0.17	0.11	0.42	-0.47	-0.27	0.16	0.77	0.64	-0.27
mhyc	0.36	-0.05	0.57	0.34	0.45	-0.16	-0.28	-0.22	0.23	0.14	-0.47	-0.14	0.04	0.28	0.39	-0.36
myoG	-0.59	-0.15	0.27	-0.01	0.35	-0.04	0.40	0.45	-0.12	0.06	-0.43	-0.51	-0.12	0.69	0.21	0.11
pcna	-0.47	-0.51	0.29	0.49	0.06	-0.36	0.54	0.57	0.37	-0.09	-0.54	0.01	0.56	0.81	0.38	-0.37
myf6	0.21	0.44	0.48	-0.30	0.53	0.62	-0.10	-0.11	-0.65	0.77	0.19	-0.82	-0.55	0.35	0.12	0.66
mstn	0.15	0.36	0.39	-0.25	0.47	0.57	-0.18	-0.05	-0.55	0.68	0.11	-0.75	-0.64	0.23	-0.03	0.62
mbnl1	0.18	-0.28	0.82	0.48	0.12	-0.04	-0.20	-0.05	0.05	0.50	-0.54	-0.38	0.14	0.52	0.55	-0.18
foxk2	-0.33	0.11	0.51	-0.06	0.50	0.09	0.46	0.32	-0.30	0.34	-0.24	-0.59	-0.20	0.74	0.40	0.19
